# The Natural Product Magnolol as a Lead Structure for the Development of Potent Cannabinoid Receptor Agonists

**DOI:** 10.1371/journal.pone.0077739

**Published:** 2013-10-30

**Authors:** Alexander Fuchs, Viktor Rempel, Christa E. Müller

**Affiliations:** PharmaCenter Bonn, Pharmaceutical Institute, Pharmaceutical Chemistry I, Bonn, Germany; University of São Paulo, Brazil

## Abstract

Magnolol (4-allyl-2-(5-allyl-2-hydroxyphenyl)phenol), the main bioactive constituent of the medicinal plant *Magnolia officinalis*, and its main metabolite tetrahydromagnolol were recently found to activate cannabinoid (CB) receptors. We now investigated the structure-activity relationships of (tetrahydro)magnolol analogs with variations of the alkyl chains and the phenolic groups and could considerably improve potency. Among the most potent compounds were the dual CB_1_/CB_2_ full agonist 2-(2-methoxy-5-propyl-phenyl)-4-hexylphenol (**61a**, *K*_i_ CB_1_∶0.00957 µM; *K*_i_ CB_2_∶0.0238 µM), and the CB_2_-selective partial agonist 2-(2-hydroxy-5-propylphenyl)-4-pentylphenol (**60**, *K*_i_ CB_1_∶0.362 µM; *K*_i_ CB_2_∶0.0371 µM), which showed high selectivity versus GPR18 and GPR55. Compound **61b**, an isomer of **61a**, was the most potent GPR55 antagonist with an IC_50_ value of 3.25 µM but was non-selective. The relatively simple structures, which possess no stereocenters, are easily accessible in a four- to five-step synthetic procedure from common starting materials. The central reaction step is the well-elaborated Suzuki-Miyaura cross-coupling reaction, which is suitable for a combinatorial chemistry approach. The scaffold is versatile and may be fine-tuned to obtain a broad range of receptor affinities, selectivities and efficacies.

## Introduction

**Figure 1:**
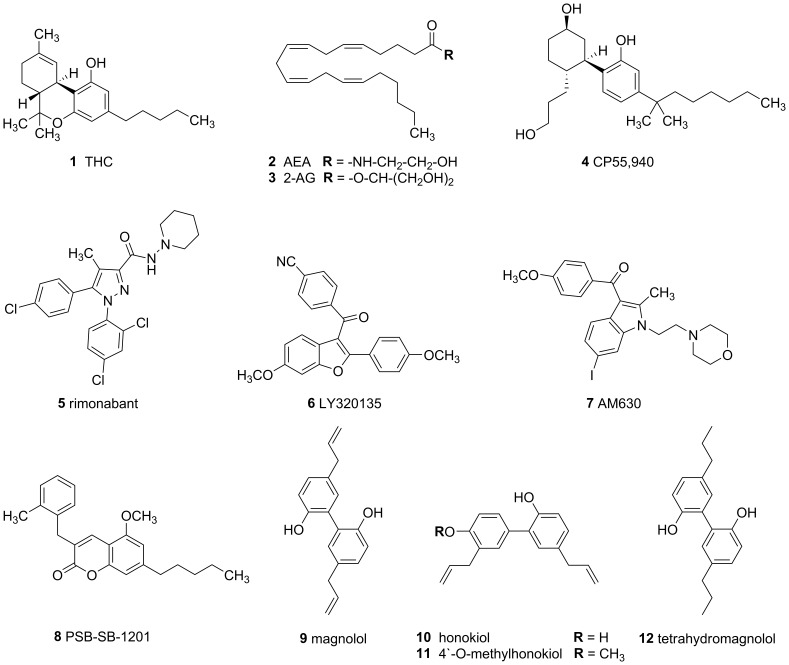
Structures of selected cannabinoid receptor ligands.

Cannabinoid (CB) receptors comprise a small family of G protein-coupled receptors (GPCR) consisting of two subtypes, CB_1_ and CB_2_. Both receptors exhibit 44% identity in amino acid sequence, are coupled to G_i/0_ proteins [1], [2], and differ in their expression patterns. The CB_1_ receptor is predominantly expressed in cells of the central nervous system, however, the CB_1_ receptor is also found in peripheral tissues like adrenal gland, bone marrow, heart, lung, prostate, testis, tonsils spleen and in adipocytes [1], [3]. The CB_2_ receptor is mainly expressed on cells of the immune system, for example in tonsils and spleen [4], [5] but it is expressed in the central nervous system as well [6].CB receptors are activated by constituents of the plant *Cannabis sativa*, e.g., Δ^9^-tetrahydrocannabinol (Δ^9^-THC, **1**) to which they owe their name. Structures of selected CB receptor ligands are shown in Figure 1. Physiological agonists, the so-called endocannabinoids, include anandamide (**2**) and 2-arachidonoylglycerol (**3**) [7].

The orphan receptor GPR55 had been proposed as a third member of the CB receptor family because of its responsiveness to some CB receptor ligands, but several studies suggested that the non-cannabinoid lysophosphatidylinositol (LPI) may be its physiological agonist [8], [9], [10]. GPR55 is expressed on cells of the central nervous system, on blood and vascular endothelial cells, bone cells, cells of the immune system and on adipocytes; it is phylogenetically distinct from both CB receptor subtypes, showing a low amino acid identity (CB_1_ 15%, CB_2_ 13%) [11].

Recently, it was discovered that several cannabinoids, including Δ^9^-THC (**1**), anandamide (**2**), and its metabolite *N*-arachidonoylglycine were agonists at the orphan receptor GPR18, which makes this receptor a likely candidate for the elusive third CB receptor subtype, despite its very low amino acid identity with the classical cannabinoid receptors (CB_1_ 12%, CB_2_ 7%) [12], [13]. The GPR18 is predominantly expressed on cells of testes and spleen, but also on thymus cells, leukocytes, in thyroid, small intestine and stomach [14], [15].

Neither potent nor selective ligands for GPR18 have been described so far [16]. Only very few moderately potent and selective ligands for GPR55 have been identified [17], [18]. In contrast, several classes of synthetic compounds have been developed that either activate CB receptors, e.g. the nonselective CB_1_/CB_2_ agonist CP55,940 (**4**), or inhibit them, e.g. the CB_1_-selective inverse agonists rimonabant (**5**), LY320135 (**6**), and the CB_2_-selective inverse agonist AM630 (**7**) [1], [19], [20]. Compounds with distinct functional properties at CB receptor subtypes have also been developed, such as PSB-SB-1201 (**8**), an antagonist at CB_1_ and an agonist at CB_2_ receptors [21]. There is widespread potential for therapeutic applications of CB receptor ligands. CB_1_ receptor agonists, for example, relieve pain, nausea and vomiting, reduce hyperexcitability in epilepsy, and increase food intake of debilitated patients. On the other hand CB_1_ receptor antagonists may be useful for the modulation of behavior in addiction and for treating obesity [22]. In fact, rimonabant (**5**) had been approved for that indication, but has meanwhile been withdrawn from the market due to serious side-effects resulting in an increased suicide rate [23]. Antinociceptive effects, especially in neuropathic and chronic pain, indications of medical need, may be achieved by selective CB_2_ receptor agonism thus avoiding adverse CB_1_ effects [24]. Further fields of application for CB_2_ receptor agonists are inflammatory diseases including multiple sclerosis and arthritis [25]. A few CB receptor agonists have already been licensed for clinical use, including dronabinol (Δ^9^-THC), nabilon, a synthetic THC analog, and a combination of Δ^9^-THC with cannabidiol to reduce psychotropic effects [24].

Since CB receptors are validated drug targets the development of novel, improved ligands is warranted. A large number of therapeutically used drugs are compounds from nature or derivatives thereof [26]. These natural products are drug-like molecules with regard to their physicochemical properties and their ability to interact with biological structures, especially with proteins, since they have been optimized throughout evolution to interact with target structures [26], [27].

Natural products have a long history of interactions with CB receptors. Preparations of the medicinal plant *Cannabis sativa* have been therapeutically used for thousands of years before their mechanism of action – the activation of CB receptors – had been discovered and the active constituents like THC (**1**) had been identified [28]. In addition to cannabis constituents further plant-derived natural products have been reported to interact with the endocannabinoid system, including the terpene *beta*-caryophyllene, fatty acid derivatives, such as *N*-linoleoylethanolamide, and various *N*-alkylamides from *Echinacea* spp. [29], [30], [31]. These compounds may either act directly on CB receptors (*beta*-caryophyllene) [29] or indirectly by inhibition of endocannabinoid degradation (*N*-linoleoylethanolamide and various *N*-alkylamides) [30],[31]. Recently we discovered that a bark extract of *Magnolia officinalis,* which has been used in traditional Chinese medicine (TCM) for the treatment of insomnia, anxiety disorders and allergic diseases [32], [33], exhibits CB-agonistic effects [34]. The main active constituents of *Magnolia officinalis* bark were shown to be the biphenylic neolignans magnolol (**9**), honokiol (**10**), and to a very minor content 4′-O-methylhonokiol (**11**) [33]. It was shown that these biphenylic compounds interact with CB receptors [34], [35]. 4′-O-Methylhonokiol (**11**), the main constituent of the seeds of *Magnolia grandiflora* L., but only a minor constituent of *Magnolia officinalis*, was found to be an inverse agonist at CB_2_ receptors with selectivity versus CB_1_ (K_i_ CB_1_ 2400 nM, K_i_ CB_2_ 43.9 nM) [35]. Extensive semisynthetic modification of **11** did not lead to any significant increase in CB_2_ receptor affinity of the natural product [35].

We recently discovered that the symmetrical biphenol magnolol (**9**) is a partial agonist at both CB receptor subtypes with K_i_ values in the low micromolar range, while its main metabolite tetrahydromagnolol (**12**) possessed 20-fold higher potency at the CB_2_ receptor along with selectivity for that subtype [34]. In the present study we therefore utilized magnolol (**9**) and its hydrogenated metabolite **12** as new lead structures for the development of potent and selective CB receptor ligands.

## Results and Discussion

### Structural Comparison

**Figure 2:**
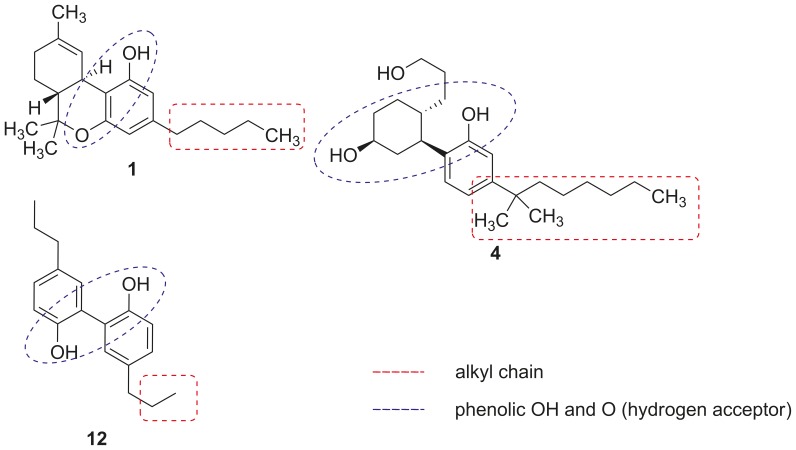
Structural comparison of Δ^9^-THC (1), the synthetic CP55,940 (4), tetrahydromagnolol (12).

Comparison of the structural features of the newly discovered partial CB_2_ receptor agonist tetrahydromagnolol (**12**) with those of the natural cannabinoid Δ^9^-THC (**1**), a partial CB_1_/CB_2_ agonist, and the synthetic CB_1_/CB_2_ full agonist CP 55,940 (**4**) showed structural similarities (Figure 2). All three structures share two directly connected six-membered rings at least one of which bears a hydroxyl group and an alkyl residue of different chain length. An additional oxygen atom – a hydrogen bond acceptor – is found in a certain distance of the hydroxyl function: in **1** it is represented by a cyclic ether structure, in **4** and **12** it constitutes a second hydroxyl group (Figure 2). Tetrahydromagnolol (**12**) is structurally much more simple than **1** and **4**. Both of the latter compounds bear multiple chiral centers, while **12** is an achiral molecule. Therefore, biphenol **12** appeared to be a very good starting point for optimization and for studying structure-activity relationships (SARs) of this new class of CB receptor ligands. We decided to keep the *o*,*o*-dihydroxybiphenyl core structure, and to (i) modify the alkyl chains to obtain symmetric as well as unsymmetric analogs with different chain lengths, and (ii) methylate one of the phenolic hydroxyl groups, a modification that has been shown to modulate CB receptor affinity and activity in other classes of CB receptor ligands, e.g. in the coumarine series represented by compound **8** (Figure 1) [21].

### Syntheses of Intermediates

**Figure 3:**
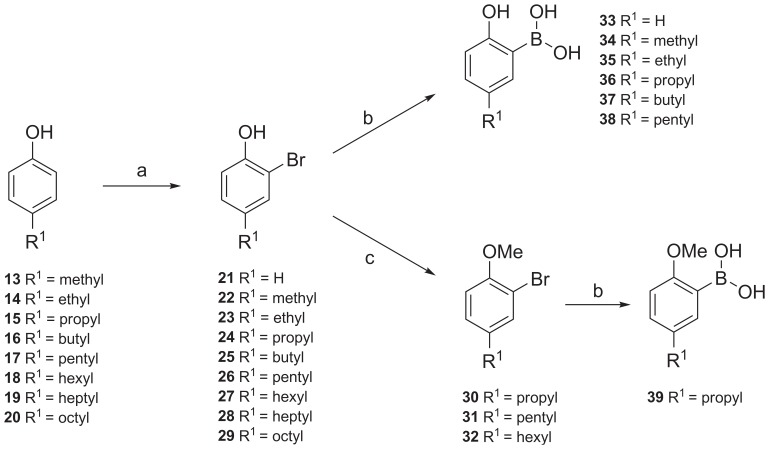
Synthesis of intermeditates. (a) Br_2_, NaHCO_3_, CHCl_3_, 0°C; (b) three steps, (1) *n*-butyllithium, Et_2_O, −78°C; (2) B(OCH_3_)_3_, Et_2_O, −78°C to rt; (3) HCl, Et_2_O; (c) CH_3_I, NaOH, benzyl-tri-*n*-butylammonium bromide, CH_2_Cl_2_ : H_2_O (1∶ 1), 12 h, rt.

Magnolol analogs were synthesized starting from different commercially available 4-alkylphenols **13**–**20** (Figure 3). The bromination of 4-alkylphenols (Figure S1) via electrophilic aromatic substitution was carried out with elementary bromine in chloroform in the presence of sodium hydrogencarbonate to give the 2-bromo-4-alkylphenols **21–29** [36]. Methylation by a phase transfer catalysis method (Figure S2) with methyl iodide as an alkylating agent in a mixture of water and dichloromethane in the presence of sodium hydroxide and benzyl-tri-*n*-butylammonium bromide led to the 2-bromo-1-methoxy-4-alkylbenzenes **30**–**32** [37]. Boronic acid derivatives **33**–**38** were obtained from the 2-bromo-4-alkylphenols **21–29** by treatment with *n*-butyllithium and trimethyl borate (Figure S3) yielding **33**–**38** after acidic hydrolysis in moderate yields [38]. 2-Methoxy-5-propylphenylboronic acid (**39**) was obtained by the same procedure.

### Syntheses of Magnolol Derivatives and Analogs

**Figure 4:**
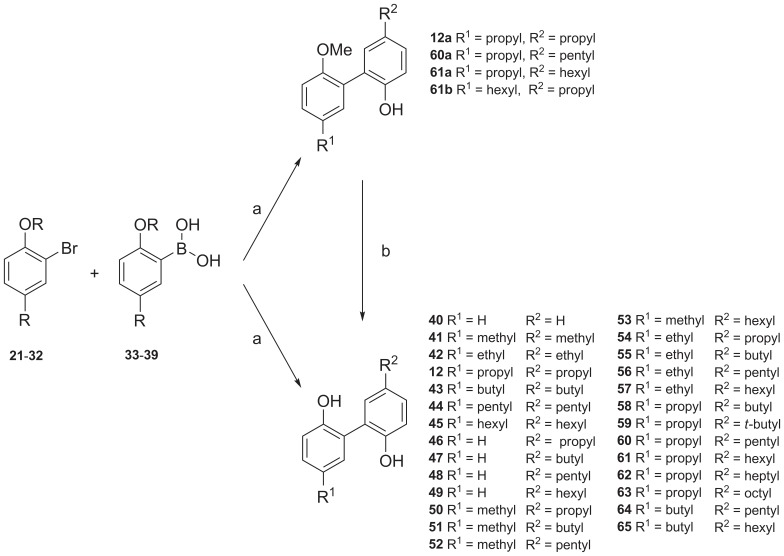
Synthesis of magnolol derivatives and analogs. (a) Pd(PPh_3_)_4_, Na_2_CO_3_, toluene, EtOH, H_2_O, 100°C, 18h; (b) CH_2_Cl_2_, BBr_3_, −78°C to rt.

The final analogs **12**, **12a**, **40**–**65**, **61a** and **61b** (Figure 4), were synthesized by Suzuki cross coupling reaction (Figure S4) [39]. We found that the coupling resulted in higher yields (ca. 65% compared to ca. 30%) when the 2-bromo-1-methoxy-4-alkylbenzenes **30**–**32** were used instead of the unmethylated 2-bromo-4-alkylphenols **21**–**29**. Demethylation [40] of **12a**, **60a**, and **61a** yielded the products **12**, **60**, and **61** (Figure S5). Overall yields when using the methylated phenols **30–32**, involving an additional demethylation step, were higher compared to the direct coupling of the 2-bromo-4-alkylphenols (ca. 50% compared to ca. 30%). The final compounds were purified by flash chromatography or HPLC, respectively. The structures were confirmed by ^1^H- and ^13^C-NMR spectra and by HPLC coupled to electrospray ionization mass spectrometry (LC-ESI-MS). The purity was confirmed by the same method and was in all cases greater than >95% (for details see Experimental Section and Supporting Information).

### Biological Evaluation

The affinities of the magnolol analogs at human CB_1_ and CB_2_ receptors were determined in radioligand binding studies using [^3^H](-)-*cis*-3-[2-hydroxy-4-(1,1-dimethylheptyl)phenyl]-*trans*-4-(3-hydroxypropyl)cyclohexanol (CP55,940, **4**) as a non-selective CB receptor radioligand. Membrane preparations of Chinese hamster ovary (CHO) cells stably expressing the respective receptor subtype were utilized as a source for human CB_1_ or CB_2_ receptors, respectively. Compounds were initially screened at a concentration of 10 µM. In cases where inhibition of radioligand binding was about 50% or more, full concentration-inhibition curves were determined in order to calculate K_i_ values. To investigate the intrinsic activities of the synthesized compounds at CB receptors cAMP accumulation assays with CHO cells stably expressing the human CB_1_ or CB_2_ receptor subtype were performed. Intracellular cAMP levels were measured by a radioactive filtration assay determining competition of [^3^H]cAMP by formed cAMP to a binding protein isolated from bovine adrenal glands [21], [41]. Effects of test compounds (10 µM) on forskolin-stimulated cAMP levels were determined relative to the maximal effect observed with the full agonist CP55,940. To determine interaction with the CB-related orphan receptors GPR18 and GPR55 β-arrestin assays with CHO cells stably expressing the respective receptor were carried out. A β-galactosidase enzyme fragment complementation technology was applied (β-arrestin PathHunter™ assay, DiscoverX, Fremont, CA, USA) to monitor β-arrestin recruitment to activated receptors. The compounds were screened for agonistic and antagonistic activity at the respective receptor at a concentration of 10 µM. Full concentration-effect curves were determined for the most potent compounds. Biological data are collected in Table 1, Table 2 and Table S1. For comparison the results of commercially available standard compounds obtained under the same assay conditions are included.

**Table 1:** Potencies and Efficacies of Magnolol Derivatives and Analogs at human Cannabinoid Receptor Subtypes.^a^

Compd.	heterologous competition vs. [^3^H]CP55,940		cAMP accumulation assay	
	CB_1_	CB_2_	CB_1_	CB_2_
	*K*_i_ (nM)		EC_50_ (nM)/efficacy	
**1**	3.88±0.91	71.6±0.024.1	6.76±3.61/88%^b^	14.0±6.8/34%^b^
**4**	1.28 [21]	1.42 [21]	2.28 [21]/100%^b^	1.00 [21]/100%^b^
**9**	3150 [34]	1440 [34]	18300/62%^c^ [34]	3280/31%^c^ [34]
**10**	6460 [34]	5610 [34]	4%^b^	0%^b^
**11**	8340±3200 (2400 [35])	43.3±17.1 (43.9 [35])	42%^b^	87%^b^
**12**	2260 [34]	416 [34]	9010/124%^c^ [34]	170/49% [34]
**12a**	267±58	221±57	622±284/112%^b^	77.8±20.5/83%^b^
**40**	>1000	>1000	n.d.	n.d.
**41**	>1000	>1000	n.d.	n.d.
**42**	2130±840	2870±770	n.d.	(−3%)
**43**	2700±1200	1590±40	7110±1430/100%^b^	378±148/67%^b^
**44**	3130±1130	833±123	4540±830/44%^b^	2300±710/62%^b^
**45**	4640±580	1830±190	−1%^b^	−14%^b^
**46**	∼ 1000	∼ 1000	n.d.	n.d.
**47**	∼ 1000	2030±880	0%^b^	31%^b^
**48**	6590±2560	1160±290	50%^b^	51%^b^
**49**	6630±5030	1500±640	74%^b^	34%^b^
**50**	>1000	7380±2760	n.d.	5%^b^
**51**	∼ 1000	1690±530	43%^b^	37%^b^
**52**	1230±470	517±101	33%^b^	37%^b^
**53**	822±224	273±96	105%^b^	42%^b^
**54**	∼ 1000	856±367	0%)^b^	12%^b^
**55**	5760±2850	235±101	88%^b^	30%^b^
**56**	634±297	161±33	91%^b^	91%^b^
**57**	386±29	83.0±11.8	114%^b^	47%^b^
**58**	3610±170	468±133	79%^b^	64%^b^
**59**	5810±2670	489±49	36%^b^	36%^b^
**60**	362±113	37.1±7.8	971±89/98%^b^	258±13/81%^b^
**60a**	17.3±1.4	31.0±9.9	37.5±5.6/95%^b^	39.9±10.0/94%^b^
**61**	145±48	**29.4**±9.0	829±278/102%^b^	159±18/70%^b^
**61a**	**9.57**±5.43	23.8±7.1	159±76/100%^b^	38.5±17/100%^b^
**61b**	313±125	281±101	**K_B_: 1850**±730/0%^b^	595±150/42%^b^

a all data resulted from three independent experiments, performed in duplicates.

b efficacy at 10 µM compared to max. effect of the full agonist CP55,940 (1 µM) = 100%.

c efficacy was determined at a concentration of 100 µM.

**Table 2:** Activities of Magnolol Derivatives and Standard Compounds at human GPR18 and GPR55.^a^

Compd.	radioligand binding assays vs. [^3^H]CP55,940		β-arrestin recruitment assay	
	CB_1_	CB_2_	GPR18	GPR55
	*K*_i_ (nM)		EC_50_ or IC_50_ (µM)	
**1**	3.88	71.6	4.61±0.50	14.2±5.4
	*agonist*	*agonist*	*agonist*	*antagonist* 65%^c^
**4**	1.28 [21]	1.42 [21]	5.99±1.88	1.61±0.47
	*agonist*	*agonist*	*antagonist* 188%^b^	*antagonist* 93%^c^
**5**	12.6 [34]	900 [34]	10.1±1.3	2.01±0.66
	*antagonist*	*antagonist*	*antagonist* 94%^b^	*agonist*
**6**	141 [47]	14900 [47]	4.97±1.51	4.14±1.05
	*antagonist*	*antagonist*	*antagonist* 81%^b^	*antagonist* 81%^c^
**7**	5150 [2], [19]	31.2 [2], [19]	∼ 10	≥10
	*agonist*	*antagonist*	*antagonist* 52%^b^	*antagonist* 48%^c^
**11**	8340	43.3	>10	7.77±0.97
	*agonist*	*agonist*	*antagonist* 37%^b^	*antagonist* 87%^c^
**12**	2260	416	30.9±15.8	13.3±2.0
	*agonist*	*agonist*	*antagonist* 64%^b^	*antagonist* 96%^c^
**12a**	267	221	>10	4.55±1.08
	*agonist*	*agonist*	*antagonist* 41%^b^	*antagonist* 82%^c^
**60**	362	37.1	14.5±2.8	>10
	*agonist*	*agonist*	*antagonist* 118%^b^	*antagonist* 45%^c^
**60a**	17.3	31.0	>10	6.93±1.06
	*agonist*	*agonist*	*antagonist* 47%^b^	*antagonist* 66%^c^
**61**	145	29.4	10.4±1.1	>10
	*agonist*	*agonist*	*antagonist* 139%^b^	*antagonist* 45%^c^
**61a**	9.57	23.8	>10	∼ 10
	*agonist*	*agonist*	*antagonist* 30%^b^	*antagonist* 58%^c^
**61b**	313	281	>10	3.25±0.29
	*antagonist*	*agonist*	*antagonist* 22%^b^	*antagonist* 120%^c^

a all data result from three independent experiments, performed in duplicates.

b % inhibition of Δ^9^-THC (10 µM)-induced β-arrestin recruitment by test compounds at a concentration of 10 µM.

c % inhibition of LPI (1 µM)-induced β-arrestin recruitment by test compounds at a concentration of 10 µM.

### Structure-Activity Relationships at CB_1_ and CB_2_ Receptors

The natural product magnolol (**9**, 4-allyl-2-(5-allyl-2-hydroxyphenyl)phenol) was recently found to show affinity for CB_1_ and CB_2_ receptors in the low micromolar range behaving as a partial agonist at both receptor subtypes [34], [35]. Its main metabolite tetrahydromagnolol (**12**), which contains two propyl instead of allyl residues due to reductive metabolization, was even more potent and showed selectivity for CB_2_ receptors [34]. Based on the (tetrahydro)magnolol (**9**, **12**) scaffold we replaced the allyl (**9**) or propyl (**12**) moieties in the *para*-position of the phenolic hydroxyl groups by a large variety of different residues ranging from hydrogen to long aliphatic alkyl chains (up to octyl). As a second modification we studied the effect of methylation of one of the phenolic hydroxyl groups in selected derivatives.

As a first step we investigated symmetrically substituted biphenyls. The simplest symmetric biphenyl derivative 2-(2-hydroxyphenyl)phenol (**40**) displayed no affinity towards CB receptors. The introduction of alkyl substituents in the R^1^ and R^2^ position markedly enhanced CB receptor affinity, with an optimum being reached by propyl substitution (**12**, *K*_i_ CB_1_∶2.26 µM, CB_2_∶0.416 µM). Longer chains (butyl (**43**), pentyl (**44**), hexyl (**45**)) resulted in decreased affinities compared to tetrahydromagnolol (**12**), emphasizing that the di-propyl substitution of the magnolol metabolite **12** was optimal for CB receptor affinity. Like the lead structures **9** and **12** the compounds of this subset of compounds displayed a preference for the CB_2_ receptor subtype. However, methylation of one of the phenolic groups of the symmetrical compound **12** led to its unsymmetrical derivative **12a** with strongly improved CB_1_ receptor affinity (*K*_i_ CB_1_∶0.267 µM, CB_2_∶0.221 µM).

To study the structure-activity relationships of magnolol analogs in more detail, we next synthesized unsymmetrically substituted biphenyls bearing diverse residues in the R^1^ and R^2^ position (also see Table S1). Due to the symmetrical structure of the biphenol core, the designation R^1^ and R^2^ is interchangeable; only in compounds where one of the phenolic groups is alkylated, the R^1^ and R^2^ positions can be distinguished. In order to facilitate the discussion of the SARs we kept the designation R^1^ and R^2^ even in the biphenolic compounds, as depicted in Figure 4. Compared to the simple biphenyl **40** (R^1^, R^2^ = H) an increase in the size of the substituent in the R^2^ position resulted in an enhanced affinity of the compounds (**46**–**49**). The determined *K*_i_ values at CB_2_ receptors increased from approximately 10 µM for the mono-propyl-substituted compound **46** to 1.16 µM for the pentyl-substituted **48**. A further elongation of the alkyl chain to hexyl (**49**) did not further improve CB_2_ receptor affinity.

By keeping the residue in the R^2^-position constant we investigated the influence of the size of the substituent on the other side (R^1^). The length of the R^1^ alkyl moiety strongly contributed to the affinity of the magnolol analogs. The rank order of potency at the CB_2_ receptor for compounds with a hexyl residue at R^2^ was as follows: R^1^ = H (**49**, *K*_i_ 1.50 µM)<methyl (**53**, 0.273 µM)<ethyl (**57**, 0.0830 µM) <propyl (**61**, 0.0294 µM). Substitution with residues larger than propyl markedly reduced affinity to CB receptors (compare R^1^ = propyl (**61**), *K*_i_ 0.0294 µM/R^1^ = butyl (**65**), 0.670 µM/R^1^ = hexyl (**45**), 1.83 µM). As a next step we kept the favorable propyl residue constant and varied the size of alkyl residues in the R^2^ position from H (**46**) to octyl (**63**). Enlargement as well as reduction of the hexyl moiety in the R^2^ position decreased affinity for CB receptors (compare **46**, **54**, **12** and **58**–**63**). Thus the optimal alkyl combination was obtained by an introduction of the medium-length propyl on the one side and the longer hexyl moiety on the other side of the biphenylic core (**61**). Compound **61** exhibited *K*_i_ values of 0.145 µM and 0.0294 µM at CB_1_ and CB_2_ receptors, respectively. The variation of the alkyl residues did not affect the preference of the compounds for CB_2_ receptors. The highest selectivity (∼25 fold) was obtained when R^1^ was substituted with an ethyl and R^2^ with a butyl residue (**55**).

The replacement of one hydroxyl by a methoxy group led to a markedly increased CB_1_ receptor affinity of the magnolol analogs, and at the same time the selectivity towards CB_2_ receptors was lost, as shown for methoxytetrahydromagnolol (**12a**). To further investigate this effect we introduced a methoxy group into the two most potent compounds of the present series (**60** and **61**) leading to the derivatives **60a**, **61a** and **61b**. Like methoxytetrahydromagnolol (**12a**) the methylated compound **60a** lost its selectivity for CB_2_ receptors; it displayed a 21-fold higher affinity towards the CB_1_ receptor compared to the parent biphenyl **60**, while CB_2_ receptor affinity was barely affected.

**Figure 5:**
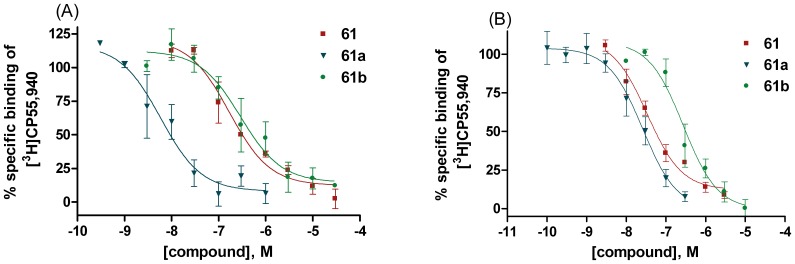
Radioligand binding results of key compounds 61, 61a, 61b. Concentration-dependent inhibition of specific [^3^H]CP55,940 binding by **61** (▪) at membrane preparations of CHO cells expressing (A) human CB_1_, or (B) human CB_2_ receptors, respectively (*K*_i_ CB_1_∶0.145 µM, CB_2_∶0.0294 µM). The biphenol **61** is substituted with two alkyl residues, a propyl residue at one side and a hexyl chain at the other side of the biphenylic core. Each alkyl side chain is located in the *para*-position of one of the phenolic hydroxyl groups. Substitution of the hydroxyl group in the *para*-position of the propyl residue (**61a** (▾)) resulted in a remarkable increase in (A) CB_1_ receptor affinity (*K*_i_: 0.00957 µM), while (B) CB_2_ receptor affinity was barely affected (*K*_i_: 0.0238 µM) compared to the parent compound **61**. An introduction of a methoxy group in *para*-position of the hexyl side chain (**61b** (•)) had different effects: (A) **61b** displayed a moderately decreased CB_1_ receptor affinity (*K*_i_: 0.313 µM) and (B) a drastical loss in CB_2_ receptor affinity (*K*_i_: 0.281 µM) compared to **61**. Data points represent means ± SEM of three independent experiments, performed in duplicates.

The position of the methoxy group had a big impact on affinity and selectivity, as demonstrated for **61** (Figure 5). The introduction of a methoxy group in the *para*-postion to the hexyl residue (**61b**) drastically reduced the affinity towards CB_2_ receptors (from a *K*_i_ of 0.0294 µM to 0.234 µM, Figure 5A), while the decrease in affinity was less pronounced at CB_1_ receptors (2-fold reduced affinity, Figure 5B). When the methoxy group was introduced in the *para*-position of the short propyl residue a remarkable boost in CB_1_ receptor affinity could be observed leading to the most potent compound of the synthesized series (**61a**). Compared to the unmethylated **61**, **61a** exhibited a 15-fold increase in CB_1_ receptor affinity (Figure 5A), while CB_2_ receptor affinity was virtually unaltered (Figure 5B). The impact of the methoxy group on CB receptor affinity was even more pronounced when the two structural isomers **61a** and **61b** were compared. While **61b** (methoxy group in the *para*-position with regard to the hexyl chain) exhibited *K*_i_ values of 0.313 µM and 0.281 µM at CB_1_ and CB_2_ receptors, respectively, **61a** (methoxy group in the *para*-position with respect to the propyl residue) displayed a 33-fold increase in CB_1_ (*K*_i_ 0.00957 µM) and 12-fold increase in CB_2_ receptor affinity (*K*_i_ 0.0238 µM).

Thus the methylation of the phenolic hydroxyl group abolished the preference of magnolol analogs for CB_2_ receptors and, depending on which of the two phenolic groups was methylated, the resulting compounds possessed an increased CB_1_ receptor affinity (**12a**, **60a**, **61a**) or decreased affinity at both receptor subtypes (**61b**) compared to the parent biphenolic compounds.

### Functional Properties

Receptor ligands may exhibit full agonistic, partial agonistic, antagonistic or inverse agonistic activity. In order to study the intrinsic activity of the new magnolol analogs at the G_i_-coupled CB_1_ and CB_2_ recepor subtypes, their inhibitory effects on forskolin-stimulated adenylate cyclase activity was determined in cAMP accumulation assays at a concentration of 10 µM, and compared to the maximal effect (set at 100%) achieved with the full CB_1_ and CB_2_ agonist CP55,940 (**4** at 1 µM). For the most potent compounds full concentration-response curves were recorded and EC_50_ values were determined. The obtained results are presented in Table 1 and in Table S1. In addition, we investigated the previously published CB_2_-selective 4′-O-methylhonokiol (**11**) for its intrinsic activity in cAMP accumulation assays [35]. While the radioligand binding results obtained in our laboratory for **11** were in accordance with the previously published data, the determined functional properties are divergent [35]. Schuehly et al. had reported inverse agonistic effects of **11** as determined in forskolin-induced cAMP accumulation assays in CHO-K1 cells stably expressing the human CB_2_ receptor. However, in our hands **11** behaved as an agonist at both CB receptor subtypes.

Since in both laboratories cell lines and the applied assay system were similar, the observed divergence in intrinsic activities may be based on different levels of constitutively active receptors in the used cell lines. For the human CB_2_ receptor it has been shown that a ligand can behave as an inverse or a partial agonist, depending on the fraction of constitutively active receptors [42], [43]. In case of a high fraction of constitutively active receptors a low efficacy ligand will behave as an inverse agonist, while the same compound can act as a partial agonist in a system with lower levels of constitutively acitve receptors [42], [43], [44]. This well described phenomenon is referred to as ‘protean agonism’ and may explain the divergent intrinsic activities for **11** observed in both laboratories [44].

**Figure 6:**
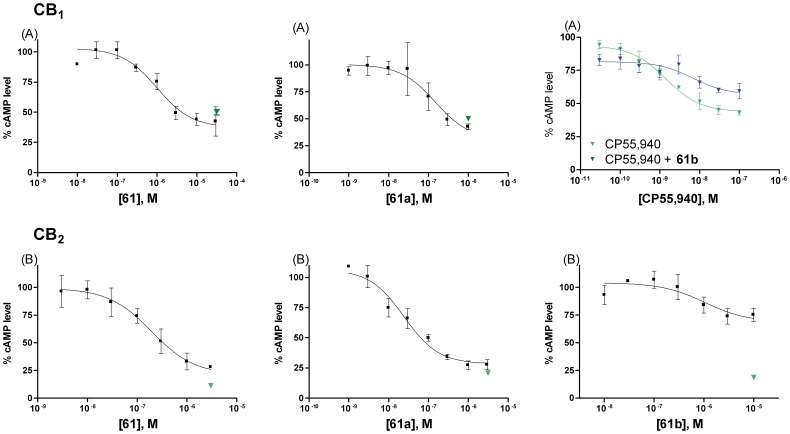
Effects of 61, 61a and 61b on forskolin(10 µM)-induced cAMP production. CHO cells expressing (A) human CB_1_, or (B) human CB_2_ receptors. The maximal effect of the full agonist CP55,940 is represented by the green triangle symbol (▾). Data points represent means ± SEMs of three independent experiments, performed in duplicates.

The naturally derived lead structure magnolol (**9**) exhibited partial agonistic activities at both receptor subtypes with somewhat higher efficacy at CB_1_ receptors than at CB_2_, but higher potency at CB_2_ receptors [34]. The compounds of the present series displayed, in general, a comparable profile. Efficacy of the synthesized compounds could be modified by variation of the alkyl chain length. The combination of a propyl residue in the R^1^-position and a pentyl (**60**) or a hexyl (**61**) moiety in the R^2^-position resulted in full agonistic effects at CB_1_ and almost full efficacy at CB_2_ receptors (Figure 6). Based on these two compounds the influence of the alkyl chain length on efficacy may be demonstrated. Residues in the R^2^-position shorter than pentyl or longer than hexyl, in combination with a propyl residue in the R^1^-position led to a partial agonistic activity at the CB_2_ receptor subtype (compare **12** and **58–63**), while efficacy at the CB_1_ receptor was barely affected (except for compound **59**). The variation of the propyl side chain in the R^1^-position, in combination with a hexyl residue in the R^2^-position also decreased CB_2_ receptor efficacy, leading to CB_2_ receptor partial agonists. Again, efficacy at the CB_1_ receptor was virtually unaltered (compare **49**, **53**, **57**, **61**, **65**). Full agonists at CB_1_ and CB_2_ receptors could be obtained by methylation of the hydroxyl group in the *para*-position to the propyl residue (**12a**, **60a**, **61a**; Figure 6). In contrast, methylation of the hydroxyl group in the *para*-position of the hexyl residue (**61b**) resulted in CB_1_-antagonistic activity and partial agonistic activity at CB_2_ receptors, emphasizing the importance of the free phenolic hydroxyl group for high intrinsic activity, i.e. efficient receptor activation by the synthesized compounds (Figure 6).

### Selectivity Towards the Related Orphan Receptors GPR18 and GPR55

It is well known that several cannabinoid receptor ligands interact with the orphan receptors GPR18 and GPR55 [12], [45]. Tetrahydromagnolol (**12**), for example, was recently identified as a weak GPR55 receptor antagonist [34]. As the physiological role of GPR18 and GPR55 is poorly understood, an interaction of newly synthesized CB ligands with these orphan receptors should be considered as it may lead to unpredictable and undesired off-target effects [46]. Thus, previously as well as newly developed CB receptor ligands should also be investigated for an interaction with these related targets. Furthermore, compounds identified to interact with one of the orphan GPCRs, might serve as useful starting point for the development of urgently needed potent and selective GPR18 or GPR55 receptor ligands.

Thus, we investigated some commercially available and broadly used CB receptor ligands as well as all of the synthesized magnolol derivatives and analogs for potential interaction with these orphan receptors (for complete results see Supporting Information). The results for the most potent CB receptor ligands of the present series as well as for standard CB receptor ligands are summarized in Table 2. All of the investigated commercially available CB receptor ligands - the nonselective agonists CP55,940 (**4**) and Δ^9^-THC (**1**), the CB_1_-selective antagonists rimonabant (**5**) and LY320135 (**6**) [47], and the CB_2_-selective antagonist AM630 (**7**) - displayed potency at least at one of the two investigated orphan receptors. CP55,940 (**4**) showed inverse agonistic activity at GPR18 and antagonistic activity at GPR55 in the low micromolar range. As already reported by others we could confirm that Δ^9^-THC (**1**) acted as an agonist at GPR18 (EC_50_ 4.61 µM) and a weak antagonist at GPR55 (IC_50_ 14.2 µM) [48], [49]. Rimonabant (**5**) showed an opposite activity profile compared to Δ^9^-THC (**1**) acting as a weak antagonist at GPR18 (IC_50_ 10.1 µM) but as an agonist at GPR55 (EC_50_ 2.01 µM) [34]. Contrary to the diarylpyrazole derivative rimonabant (**5**), the benzofurane derivative and CB_1_ receptor antagonist LY320135 (**6**) displayed antagonism at both orphan receptors with IC_50_ values in low micromolar range (IC_50_ GPR18∶4.97 µM; IC_50_ GPR55∶4.14). The CB_2_-selective antagonist AM630 (**7**), an indole derivative, was a weak antagonist at GPR18 and GPR55 displaying IC_50_ values in the range of 10 µM (Table 2).

None of the compounds of the magnolol-derived series showed agonistic activity at the investigated orphan receptors GPR18 and GPR55 (see Table S2). But some of the synthesized compounds inhibited Δ^9^-THC (10 µM)-mediated β-arrestin recruitment at GPR18 at a test concentration of 10 µM. In the presence of these compounds the measured β-arrestin recruitment was even lower than the basal level, indicating that these compounds acted as inverse agonists. However, concentration-response curves revealed only moderate antagonistic potency (IC_50_≥10 µM) of the compounds. The compound with the highest antagonistic potency turned out to be **61**. Due to limited solubility of the compound the antagonistic potency could only by estimated by extrapolation of the concentration-response curve (estimated IC_50_ value: 10.4 µM). Thus **61** still exhibited an approximately 70-fold selectivity for CB_1_ and 350-fold selectivity for the CB_2_ receptor subtype versus GPR18 (Table 2).

Most of the newly synthesized compounds failed to interact with GPR55. However antagonistic potency at GPR55 was found to be markedly increased by methylation of one of the hydroxyl groups (compare **12**, **12a**; **60**, **60**a and **61**, **61a**, **61b**) (see Table 2). In particular methylation of the phenolic hydroxyl group in the *para*-position with regard to the hexyl residue led to the potent GPR55 antagonist **61b** with an IC_50_ value of 3.25 µM, representing a potential new starting point for the development of GPR55 receptor antagonists with improved potency and selectivity.

## Conclusions

Based on the natural product magnolol, that was recently discovered to activate CB receptors, we designed and synthesized a series of analogs, and tested them in radioligand binding studies and cAMP accumulation assays at CB_1_ and CB_2_ receptors. Compared to the lead structures magnolol and tetrahydromagnolol a more than 230-fold increase in CB_1_ and a greater than 17-fold increase in CB_2_ receptor affinity could be achieved. Like the lead structure almost all of the newly synthesized compounds possessed agonistic activity at CB receptors, exhibiting higher efficacy at CB_1_ than at CB_2_, but higher potency at CB_2_ as compared to the CB_1_ receptor subtype. Potency and efficacy could easily be altered by methylation of one of the phenolic hydroxyl groups (compare **12**/**12a**, **60**/**60a** and **61**/**61a**/**61b**). Depending on the position of the methoxy group full agonists at both receptors (**61a**), or compounds with antagonistic activity at CB_1_ and partial agonistic activity at CB_2_ could be obtained (**61b**), thereby emphasizing the versatility of the biphenyl scaffold for the development of CB receptor ligands.

All compounds were tested for activity at the related orphan receptors GPR18 and GPR55 to investigate their selectivity, since many commercially available CB ligands were shown to interact with those orphan receptors. We could demonstrate that the frequently used standard CB ligands **1**, **4**, **5**, **6** and **7** also interact with the orphan receptors GPR18 and GPR55. Consequently, these finding will limit their suitability as pharmacological tools. In contrast, the new magnolol analogs were found to be, in general, highly selective for CB receptors, as they showed no or only moderate inhibition of GPR18 and GPR55, while none of the compounds were able to activate the orphan receptors. Because of their selectivity for CB receptors over the orphan receptors GPR18 and GPR55 some of the presented compounds can be considered as unique in the class of CB receptor ligands. However we could demonstrate that minor modifications such as the methylation of a phenolic hydroxyl group, could increase inhibitory potency at GPR55. Compound **61b** was the most potent GPR55 antagonist of the present series with an IC_50_ value of 3.25 µM, but it was even more potent at the CB receptors (*K*_i_ CB_1_∶0.313 µM; *K*_i_ CB_2_∶0.281 µM). Further optimization of this class of compounds towards GPR55 antagonistic activity may lead to the development of potent and selective GPR55 antagonists.

We developed CB agonists with selectivity for CB_2_ receptors such as 2-(2-hydroxy-5-propyl-phenyl)-4-pentylphenol (**60**, *K*_i_ CB_1_∶0.362 µM; *K*_i_ CB_2_∶0.0371 µM) and 2-(2-hydroxy-5-propyl-phenyl)-4-hexylphenol (**61**, *K*_i_ CB_1_∶0.145 µM; *K*_i_ CB_2_∶0.0294 µM). Both compounds were full agonists at CB_1_, and partial agonists at CB_2_ receptors (efficacy 60∶81%, 61∶70%). Furthermore, dual CB_1_/CB_2_ full agonists with high potency were obtained, including 2-(2-methoxy-5-propyl-phenyl)-4-pentylphenol (**60a**, *K*_i_ CB_1_∶0.0173 µM; *K*_i_ CB_2_∶0.0310 µM) and 2-(2-methoxy-5-propyl-phenyl)-4-hexylphenol (**61a**, *K*_i_ CB_1_∶0.00957 µM; *K*_i_ CB_2_∶0.0238 µM). The relatively simple structures, which possess no stereocenters, are easily accessible in a four- to five-step synthetic procedure from common starting materials. The central reaction step is the well-elaborated Suzuki-Miyaura cross-coupling reaction, which is suitable for a combinatorial chemistry approach. Due to their favourable properties further investigation in animal studies is warranted.

## Materials and Methods

All commercially available reagents were obtained from various producers (Acros, Aldrich, Fluka, Merck, and Sigma) and used without further purification. Compounds **13–21**, **30**, and **40** were commercially available. Solvents were used without additional purification or drying, unless otherwise noted. The used petroleum ether had boiling point between 40 and 80°C. The reactions were monitored by thin layer chromatography (TLC) using aluminum sheets with silica gel 60 F_254_ (Merck). Column chromatography was carried out with silica gel 0.060–0.200 mm, pore diameter ca. 6 nm. Purity of compounds was determined by LC-MS by recording mass spectra on an API 2000 (Applied Biosystems, Darmstadt, Germany) mass spectrometer (turbo ion spray ion source) coupled with a Waters HPLC system (Agilent 1100) using a Phenomenex Luna 3μ C18 column. Purity of all tested compounds was ≥95% unless otherwise noted. ^1^H- and ^13^C-NMR spectra were recorded on a Bruker Avance 500 MHz spectrometer. CDCl_3_, DMSO-d_6_, MeOD-d_4,_ or D_2_O were used as solvents as indicated below. Shifts are given in ppm relative to the remaining protons of the deuterated solvents used as internal standard (^1^H, ^13^C). Melting points were determined on the Büchi melting point apparatus B-545 and are uncorrected.

### Syntheses

4′-O-Methylhonokiol (**11**) was synthesized (Figure S6) as described before [35]. The syntheses of compounds **22–35** have previously been described and were conducted according to published procedures. For details see Supporting Information. Magnolol derivatives **12** and **41–45** have previously been described in the literature but were prepared according to a new procedure described herein. Compounds **36–39** as well as magnolol analogs **46–65** and **12a**, **60a**, **61a** and **61b** are new compounds.

### General Procedure for the Synthesis of Boronic Acid Derivatives 34–39

A solution of *n*-butyllithium (1.7 M in hexane, 38 mL) was slowly added to a cooled (–80°C) solution of 30 mmol 2-bromo-4-alkylphenol or 30 mmol of 2-bromo-1-methoxy-4-alkylphenol respectively, in dry ether (80 mL). The mixture was then allowed to warm up and stirred at rt for 2 h under an argon atmosphere. It was then cooled again (–80°C) and trimethyl borate (5.58 mL, 50 mmol) was rapidly added. The mixture was stirred at –80°C for 0.5 h and then at rt for 15 h under an argon atmosphere. Then 20 mL of 2 M aq. HCl solution were added slowly into the ice-cold reaction mixture and the mixture was stirred again for 0.5 h, while the milky white emulsion gradually became clear. The ethereal layer was then separated and the aqueous layer was extracted with diethyl ether (3 times with 100 mL each). The combined ether solutions were dried (MgSO_4_) and after filtration the solvent was evaporated under reduced pressure. The residual solid was recrystallized from hot diethyl ether : toluene, 3∶7) to give a white solid.

### General Procedure for the Synthesis of Magnolol Analogs 12, 12a, 41–65, 60a, 61a, 61b

A solution of toluene (25 mL), ethanol (5 mL) and water (5 mL) in a pressure flask was flushed with argon. While keeping a positive pressure of argon 42 mmol of boronic acid, 42 mmol of 2-bromo-4-alkylphenol or 42 mmol of 2-bromo-1-methoxy-4-alkylphenol respectively, 12.3 mmol (1300 mg) of Na_2_CO_3_ and 0.108 mmol (125 mg) of tetrakis(triphenylphosphine)palladium(0) were added. The pressure flask was closed and the mixture was stirred for 18 h at 100°C. The aqueous layer was then separated and extracted three times with ethyl acetate (80 mL portions each). The combined organic extracts were evaporated under reduced pressure. The final workup of the residue was done by column chromatography (petroleum ether : ethyl acetate = 9∶ 1).

### General Procedure for the Demethylation of Magnolol Analogs 12a, 60a, 61a, 61b to give 12, 60, 61

A solution of 14 mmol methylated magnolol analog in dry dichloromethane (60 mL) under an argon atmosphere was cooled to −80°C. While the solution was stirred constantly, 15 mmol of BBr_3_ (15 ml of a 1 M solution in hexane) was added. The solution was stirred for 1.5 h at −80°C and then allowed to warm up to 0°C. At 0°C 120 mL of water were added. The aqueous layer was then separated and extracted three times with dichloromethane (50 mL portions each). The combined organic extracts were evaporated under reduced pressure. The final workup of the residue was done by column chromatography (petroleum ether : ethyl acetate = 9∶ 1).

#### 5-Pentyl-5′-propylbiphenyl-2,2′-diol (60)

^1^H NMR (500 MHz, CDCl_3_) δ 7.12 (dd, *J* = 8.2, 2.2 Hz, CH_ar_, 2H), 7.08 (d, *J* = 2.1 Hz, CH_ar_, 2H), 6.94 (dd, *J* = 8.2, 1.1 Hz, CH_ar_, 2H), 5.58 (s, OH, 2H), 2.57 (m, ar-CH_2_, 4H_,_), 1.68–1.58 (m, CH_2_, 4H), 1.38–1.29 (m, CH_2_-CH_2_, 4H), 0.96 (t, *J* = 7.3 Hz,CH_3_, 3H), 0.90 (t, *J* = 7.0 Hz, CH_3_, 3H). ^13^C NMR (126 MHz, CDCl_3_) δ 150.74 (C_ar_-O), 150.70 (C_ar_-O), 136.04 (C_ar_), 135.77 (C_ar_), 130.97 (C_ar_), 130.90 (C_ar_), 129.70 (C_ar_), 129.65 (C_ar_), 123.59 (C_ar_), 116.39 (C_ar_), 37.13 (ar-CH_2_), 35.01 (ar-CH_2_), 31.47 (CH_2_), 31.32 (CH_2_), 24.69 (CH_2_), 22.49 (CH_2_), 14.00 (CH_3_), 13.78 (CH_3_). LC/ESI-MS (negative mode) m/z 297 (M-H)^−^, 100% (Figure S7). Yield 26.3%.

#### 5-Hexyl-5′-propylbiphenyl-2,2′-diol (61)

^1^H NMR (500 MHz, CDCl3) δ 7.11 (dd, J = 8.2, 2.2 Hz, CH_ar_, 2H), 7.08 (d, J = 2.2 Hz, CH_ar_, 2H), 6.93 (dd, J = 8.2, 1.6 Hz, CH_ar_, 2H), 5.55 (s, OH, 2H), 2.61–2.54 (m, ar-CH_2_, 4H), 1.69–1.57 (m, CH_2_, 4H), 1.38–1.28 (m, CH_2_-CH_2_-CH_2_, 6H), 0.96 (t, J = 7.3 Hz, CH_3_, 3H), 0.89 (t, J = 7.0 Hz, CH_3_, 3H). ^13^C NMR (126 MHz, CDCl_3_) δ 150.72 (C_ar_-O), 150.68 (C_ar_-O), 136.01 (C_ar_), 135.74 (C_ar_), 131.02 (C_ar_), 130.95 (C_ar_), 129.62 (C_ar_), 129.57 (C_ar_), 123.81 (C_ar_, 2C), 116.42 (C_ar_, 2C), 37.14 (ar-CH_2_), 35.05 (ar-CH_2_), 31.67 (CH_2_), 31.61 (CH_2_), 28.95 (CH_2_), 24.69 (CH_2_), 22.57 (CH_2_), 14.04 (CH_3_), 13.79 (CH_3_). LC/ESI-MS (negative mode) m/z 311 (M-H)^−^, 100% (Figure S8). Yield 27.9%.

#### 5-Hexyl-2′-methoxy-5′-propylbiphenyl-2-ol (61a)

^1^H NMR (500 MHz, CDCl3) δ 7.19 (dd, J = 8.3, 2.3 Hz, CH_ar_ 1H), 7.16 (d, J = 2.2 Hz, CH_ar_ 1H), 7.11 (dd, J = 8.2, 2.2 Hz, CH_ar_ 1H), 7.07 (d, J = 2.2 Hz, CH_ar_ 1H), 6.98–6.94 (m, CH_ar_, 2H), 6.27 (s, OH, 1H), 3.88 (s, O-CH_3_, 3H), 2.61 (t, J = 7.5 Hz, ar-CH_2_, 2H), 2.59 (t, J = 7.5 Hz, ar-CH_2_, 2H), 1.70–1.59 (m, CH_2_, 4H), 1.40–1.28 (m, CH_2_-CH_2_-CH_2_, 6H), 0.96 (t, J = 7.3 Hz, CH_3_, 3H), 0.90 (t, J = 7.0 Hz, 3H). ^13^C NMR (126 MHz, CDCl3) δ 153.50 (C_ar_-O), 151.65 (C_ar_-O), 136.45 (C_ar_), 135.32 (C_ar_), 132.49 (C_ar_), 131.02 (C_ar_), 129.04 (C_ar_), 128.89 (C_ar_), 127.12 (C_ar_), 126.17 (C_ar_), 117.30 (C_ar_), 111.47 (C_ar_), 56.31 (O-CH_3_), 37.17 (ar-CH_2_), 35.16 (ar-CH_2_), 31.74 (CH_2_), 31.67 (CH_2_), 29.04 (CH_2_), 24.71 (CH_2_), 22.63 (CH_2_), 14.09(CH_3_), 13.81 (CH_3_). LC/ESI-MS (negative mode) m/z 325 (M-H)^−^, (positive mode) m/z 327 (M+H)^+^, 100% (Figure S9). Yield 35.2%.

#### 5′-Hexyl-2′-methoxy-5-propylbiphenyl-2-ol (61b)

^1^H NMR (500 MHz, CDCl_3_) δ 7.19 (dd, *J* = 8.3, 2.2 Hz, CH_ar_, 1H), 7.16 (d, *J* = 2.2 Hz, CH_ar_, 1H), 7.11 (dd, *J* = 8.2, 2.2 Hz, CH_ar_, 1H), 7.08 (d, *J* = 2.2 Hz, CH_ar_, 1H), 6.97 (d, *J* = 3.5 Hz, CH_ar_, 1H), 6.95 (d, *J* = 3.4 Hz, CH_ar_, 1H), 6.28 (s, OH, 1H), 3.88 (s, O-CH_3_, 3H), 2.62 (t, *J* = 7.5 Hz, ar-CH_2_, 2H), 2.58 (t, *J* = 7.5 Hz, ar-CH_2_, 2H), 1.71–1.59 (m, CH_2_-CH_2_, 4H), 1.40–1.29 (m, CH_2_-CH_2_-CH_2_, 6H), 0.97 (t, *J* = 7.3 Hz, CH_3_, 3H), 0.90 (t, *J* = 6.9 Hz, CH_3_, 3H). ^13^C NMR (126 MHz, CDCl_3_) δ 153.46 (C_ar_-O), 151.70 (C_ar_-O), 136.70 (C_ar_), 135.07 (C_ar_), 132.42 (C_ar_), 131.09 (C_ar_), 129.09 (C_ar_), 128.83 (C_ar_), 127.11 (C_ar_), 126.16 (C_ar_), 117.28 (C_ar_), 111.48 (C_ar_), 56.30 (O-CH_3_), 37.29 (ar-CH_2_), 35.07 (ar-CH_2_), 31.70 (CH_2_), 31.62 (CH_2_), 28.96 (CH_2_), 24.76 (CH_2_), 22.61 (CH_2_), 14.08 (CH_3_), 13.89 (CH_3_). LC/ESI-MS (negative mode) m/z 325 (M-H)^−^, (positive mode) m/z 327 (M+H)^+^, 100% (Figure S10). Yield 61.1%.

### Retroviral Transfection

CHO K1 cells stably transfected with the human CB_1_ and CB_2_ receptor were generated with a retroviral transfection system as previously described [21]. 48 h after transfection, cells were selected by adding 0.8 mg/ml of G418 to the cell culture medium (DMEM/F12 supplemented with 10% FCS, 100 U/ml penicillin, 100 µg/ml streptomycin). After one week the G418 concentration was reduced to 0.2 mg/ml.

### Cell Culture

GP+envAM12 packaging cells were cultured at 37°C, 5% CO_2_ in HXM medium which consists of DMEM, 10% FCS, 100 U/ml penicillin G, 100 µg/ml streptomycin, 1% ultraglutamine, 0.2 mg/ml hygromycin B, 15 µg/ml hypoxanthine, 250 µg/ml xanthine and 25 µg/ml mycophenolic acid. CHO K1 cells were maintained in DMEM/F12 medium with 10% FCS, 100 U/ml penicillin, 100 µg/ml streptomycin under the same conditions. CHO cells stably transfected with the human CB_1_ and CB_2_ receptors were maintained at 37°C and 5% CO_2_ in the same medium, however in the presence of 0.2 mg/ml G418.

### Membrane Preparations for CB Receptor Assays

Membranes of CHO cells expressing the respective human CB receptor subtype were prepared as previously described [21] The obtained membrane pellets were resuspended and homogenized in the required amount of 50 mM Tris-HCl puffer, pH 7.4, to obtain a protein concentration of 5–7 mg/mL. Aliquots of the membrane preparation (1 mL each) were stored at −80°C until used.

### Radioligand Binding Assays at CB_1_ and CB_2_ Receptors

Competition binding assays were performed as described elsewhere using the CB agonist radioligand [^3^H](-)-*cis*-3-[2-hydroxy-4-(1,1-dimethylheptyl)phenyl]-*trans*-4-(3-hydroxypropyl) cyclohexanol (CP55,940, **4**, (final concentration 0.1 nM) [21]. As a source for human CB_1_ and CB_2_ receptors membrane preparations of Chinese hamster ovary (CHO) cells stably expressing the respective receptor subtype were used (25 µg of protein per vial for CB_1_ assays, and 1 µg of protein per vial for CB_2_ receptor assays, respectively). Stock solutions of the test compound were prepared in DMSO. The final DMSO concentration in the assay was 2.5%. Data were obtained from three independent experiments, performed in duplicates. Data were analyzed using Graph Pad Prism Version 4.02 (San Diego, CA, USA). For the calculation of K_i_ values the Cheng-Prusoff equation and a K_D_ value of 2.4 nM ([^3^H]CP55,940 at hCB_1_) and 0.7 nM ([^3^H]CP55,940 at hCB_1_) were used.

### cAMP Accumulation Assays

Inhibition of adenylate cyclase activity was determined in CHO cells stably expressing the CB_1_ or the CB_2_ receptor subtype, respectively, using a competition binding assay for cAMP according to the procedure described before [21]. Data were obtained from three independent experiments, performed in duplicates. Data were analyzed using Graph Pad Prism Version 4.02 (San Diego, CA, USA).

### β-arrestin Recruitment Assays

Interaction with the GPR18 and the GPR55 was investigated by performing β-arrestin assays, based on β-galactosidase enzyme fragment complementation technology (β-arrestin PathHunter™ assay, DiscoverX, Fremont, CA, USA). Therefore CHO cells stably expressing the respective receptor were seeded in a volume of 90 µL and a density of 20,000 cells/well into a 96-well plate and were incubated for 24 h at 37°C in assay medium (Opti-MEM, 2% fetal calf serum (FCS), 100 U/mL penicillin, 100 µg/mL Streptomycin, 800 µg/mL geneticin und 300 µg/mL hygromycin). After preincubation, test compounds were diluted in phosphate buffered saline (PBS buffer) containing 10% DMSO and 0.1% BSA and added to the cells in a volume of 10 µL, followed by an incubation for 90 min at 37°C. For determination of baseline luminescence PBS buffer (containing 10% DMSO, 0.1% BSA) in the absence of test compound was used. During the incubation period, the detection reagent was prepaired. For determination of β-arrestin recruitment to GPR18 the provided detection reagent was used, according to the suppliers protocol. The detection reagent for GPR55 was varied and obtained by mixing the chemiluminescent substrate Galacton-Star® (2 mM), with the luminescence enhancer Emerald-II™ and a lysis buffer (10 mM TRIS, 1 mM EDTA, 100 mM NaCl, 5 mM MgCl_2_, 1% Triton-X; pH 8) in a ratio of 1∶5:19. After the addition of 50 µL/well of detection reagent the measurement plate was incubated for further 60 min at room temperature. Finally luminescence was determined in a luminometer (TopCount NXT, Packard/Perkin-Elmer).

For the determination of antagonistic properties of test compounds the assay was performed as described for agonists, except that the test compounds were added to the cells in a volume of 5 µL/well 60 min prior to addition of the agonist (lysophosphatidylinositol = LPI, 5 µL/well).

Data were obtained from three independent experiments, performed in duplicates. Data were analyzed using Graph Pad Prism Version 4.02 (San Diego, CA, USA).

## Supporting Information

Figure S1 **Bromination of** ***para*** **-substituted phenols.** To a solution of 4-alkylphenol (20 mmol) in chloroform (20 mL), sodiumhydrogencarbonate (2 g, 24 mmol) was added. The resulting suspension was cooled to 0°C. While a solution of elementary bromine (1.12 mL, 22 mmol) in chloroform (8 mL) was slowly added, the suspension was vigorously stirred. After completion of the reaction, monitored by TLC the suspension was filtered. The filter with the solid residue was rinsed once with 50 mL of chloroform. The combined organic solutions were evaporated under reduced pressure. The final workup of the product was done either by distillation or by column chromatography (petroleum ether : ethyl acetate, 9∶ 1).
(TIF)

Figure S2 **Methylation of 2-bromo-4-alkylphenols.** A mixture of dichloromethane (50 mL), water (50 mL), phenol (10 mmol), sodium hydroxide (0.6 g, 15 mmol), methyl iodide (1.87 mL, 30 mmol) and benzyl tri-*n*-butylammonium bromide (0.36 g, l mmol) was stirred vigorously at rt for 12 h. The organic layer was then separated and the aq. layer extracted twice with dichloromethane (30 mL portions each). The combined organic extracts were evaporated under reduced pressure. The final workup of the residue was done by column chromatography (petroleum ether : ethyl acetate, 9∶ 1).
(TIF)

Figure S3 **Synthesis of boronic acid derivatives.** A solution of *n*-butyllithium (1.7 M in hexane, 38 mL) was slowly added to a cooled (–80°C) solution of 30 mmol 2-bromo-4-alkylphenol or 30 mmol of 2-bromo-1-methoxy-4-alkylphenol respectively, in dry ether (80 mL). The mixture was then allowed to warm up and stirred at rt for 2 h under an argon atmosphere. It was then cooled again (–80°C) and trimethyl borate (5.58 mL, 50 mmol) was rapidly added. The mixture was stirred at –80°C for 0.5 h and then at rt for 15 h under an argon atmosphere. Then 20 mL of 2 M aq. HCl solution were added slowly into the ice-cold reaction mixture and the mixture was stirred again for 0.5 h, while the milky white emulsion gradually became clear. The ethereal layer was then separated and the aqueous layer was extracted with diethyl ether (3 times with 100 mL each). The combined ether solutions were dried (MgSO_4_) and after filtration the solvent was evaporated under reduced pressure. The residual solid was recrystallized from hot diethyl ether : toluene, 3∶7) to give a white solid.
(TIF)

Figure S4 **Suzuki cross-coupling.** A solution of toluene (25 mL), ethanol (5 mL) and water (5 mL) in a pressure flask was flushed with argon. While keeping a positive pressure of argon 42 mmol of boronic acid, 42 mmol of 2-bromo-4-alkylphenol or 42 mmol of 2-bromo-1-methoxy-4-alkylphenol respectively, 12.3 mmol (1300 mg) of Na_2_CO_3_ and 0.108 mmol (125 mg) of tetrakis(triphenylphosphine)palladium(0) were added. The pressure flask was closed and the mixture was stirred for 18 h at 100°C. The aqueous layer was then separated and extracted three times with ethyl acetate (80 mL portions each). The combined organic extracts were evaporated under reduced pressure. The final workup of the residue was done by column chromatography (petroleum ether : ethyl acetate = 9∶ 1).
(TIF)

Figure S5 **Demethylation.** A solution of 14 mmol of methylated magnolol analog in dry dichloromethane (60 mL) under an argon atmosphere was cooled to −80°C. While the solution was stirred constantly, 15 mmol of BBr_3_ (15 ml of a 1 M solution in hexane) was added. The solution was stirred for 1.5 h at −80°C and then allowed to warm up to 0°C. Then 120 mL of water were added while the solution was at 0°C. The aqueous layer was then separated and extracted three times with dichloromethane (50 mL portions each). The combined organic extracts were evaporated under reduced pressure. The final workup of the residue was done by column chromatography (petroleum ether : ethyl acetate = 9∶ 1).
(TIF)

Figure S6 **Synthesis of 4′-O-methylhonokiol.** Me_2_SO4 (17 µL, 0.18 mmol) was added to a solution of honokiol (40 mg, 0.15 mmol) in an aqueous KOH solution (5 mL, 10%) and stirred for 1 h at 95°C. After cooling to rt HCl (1 M, 0.5 mL) was added and the mixture was subsequently extracted with chloroform (5 mL portions each). The organic layers were dried over Na_2_SO4 and after filtration they were concentrated under reduced pressure. The residue was subjected to HPLC separation (see below).
(TIF)

Figure S7 **LC/ESI-MS spectrum of 60 (mass spectrum in the positive and negative mode), HPLC chromatogram (HPLC-DAD measured from 220–400 nm) of 60, and its purity determined by HPLC-DAD from 220–400 nm (100%).**
(TIF)

Figure S8 **LC/ESI-MS spectrum of 61 (mass spectrum in the positive and negative mode), HPLC chromatogram (HPLC-DAD measured from 220–400 nm) of 61, and its purity determined by HPLC-DAD from 220–400 nm (100%).**
(TIF)

Figure S9 **LC/ESI-MS spectrum of 61a (mass spectrum in the positive and negative mode), HPLC chromatogram (HPLC-DAD measured from 220–400 nm) of 61a, and its purity determined by HPLC-DAD from 220–400 nm (100%).**
(TIF)

Figure S10 **LC/ESI-MS spectrum of 61b (mass spectrum in the positive and negative mode), HPLC chromatogram (HPLC-DAD measured from 220–400 nm) of 61b, and its purity determined by HPLC-DAD from 220–400 nm (100%).**
(TIF)

Table S1 **Potencies and Efficacies of Magnolol Derivatives and Analogs at human Cannabinoid Receptor Subtypes^a^.** ^a^all data resulted from three independent experiments, performed in duplicates. ^b^efficacy at 10 µM compared to max. effect of the full agonist CP55,940 (1 µM) = 100%. ^c^efficacy was determined at a concentration of 100 µM. ^d^% inhibition of radioligand binding at 10 µM. ^e^nd = not determined.
(DOCX)

Table S2 **Activities of magnolol analogs and standard cannabinoid receptor ligands at human GPR18 and GPR55^a^.** ^a^ all data result from three independent experiments, performed in duplicates. ^b^effect of test compounds (10 µM) on β-arrestin recruitment at human GPR18 is related to the effect of Δ^9^-THC in a concentration of 10 µM = 100%. ^c^effect of test compounds (10 µM) on β-arrestin recruitment at human GPR55 is related to the effect of LPI in a concentration of 1 µM = 100%. ^d^n.d. = not determined.
(DOCX)

Dataset S1 **Analytical data and yields of synthesized compounds.**
(DOCX)

Dataset S2 **Alignment of amino acid sequences.** Applied Software: Clustal W2 provided by European Molecular Biology Laboratory - European Bioinformatics Institute (EMBL-EBI) (http://www.ebi.ac.uk/Tools/msa/clustalw2/).
(DOCX)
