# Supplementary figures and images for: The Natural Product Magnolol as a Lead Structure for the Development of Potent Cannabinoid Receptor Agonists
Source: PLoS One. 2013 Oct 30;8(10):e77739. doi: 10.1371/journal.pone.0077739 (PMC3813752; doi:10.1371/journal.pone.0077739)

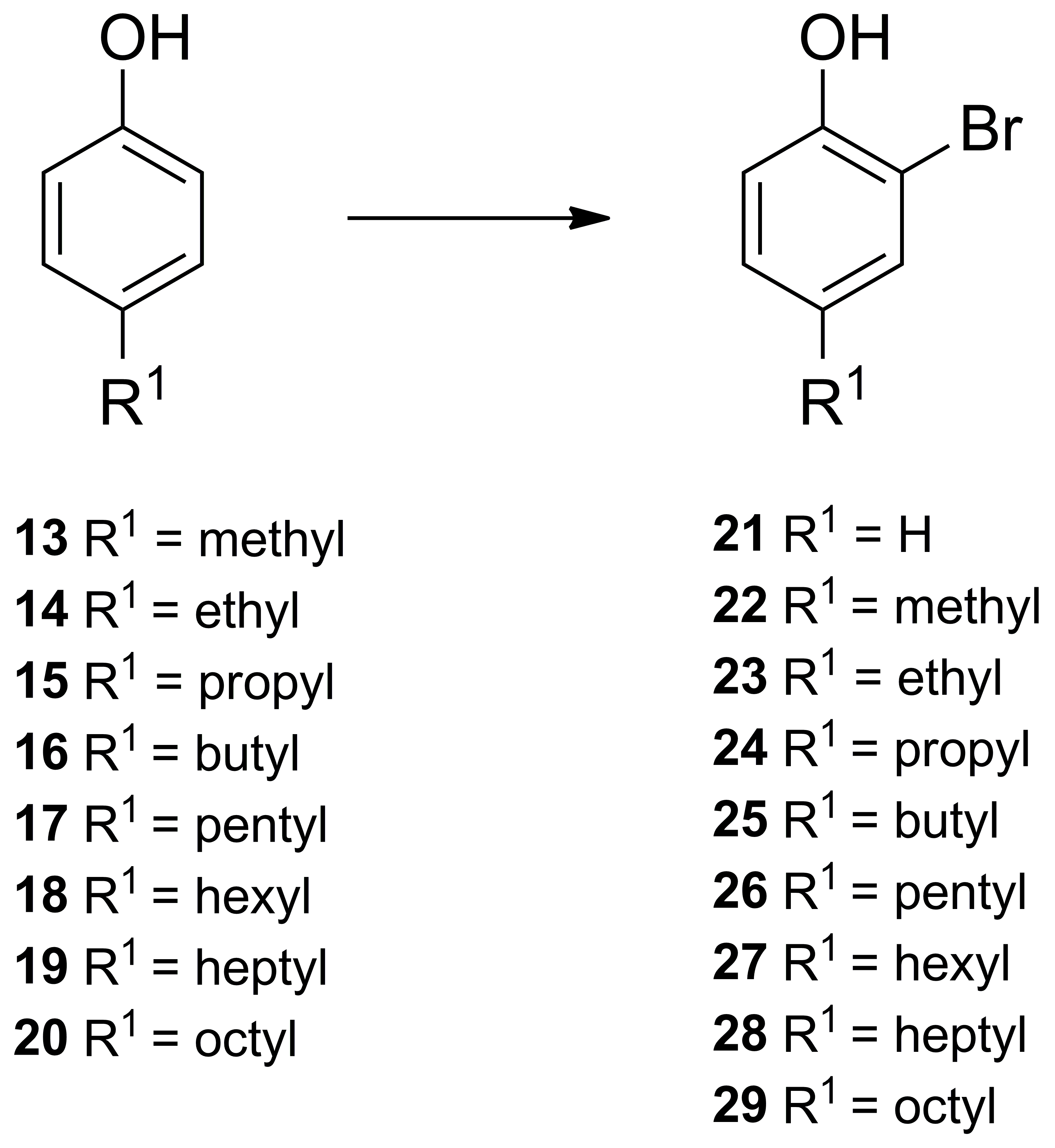

Supplement: Figure S1 — Bromination of para -substituted phenols. To a solution of 4-alkylphenol (20 mmol) in chloroform (20 mL), sodiumhydrogencarbonate (2 g, 24 mmol) was added. The resulting suspension was cooled to 0°C. While a solution of elementary bromine (1.12 mL, 22 mmol) in chloroform (8 mL) was slowly added, the suspension was vigorously stirred. After completion of the reaction, monitored by TLC the suspension was filtered. The filter with the solid residue was rinsed once with 50 mL of chloroform. The combined organic solutions were evaporated under reduced pressure. The final workup of the product was done either by distillation or by column chromatography (petroleum ether : ethyl acetate, 9∶ 1). (TIF) [file pone.0077739.s001.tif]

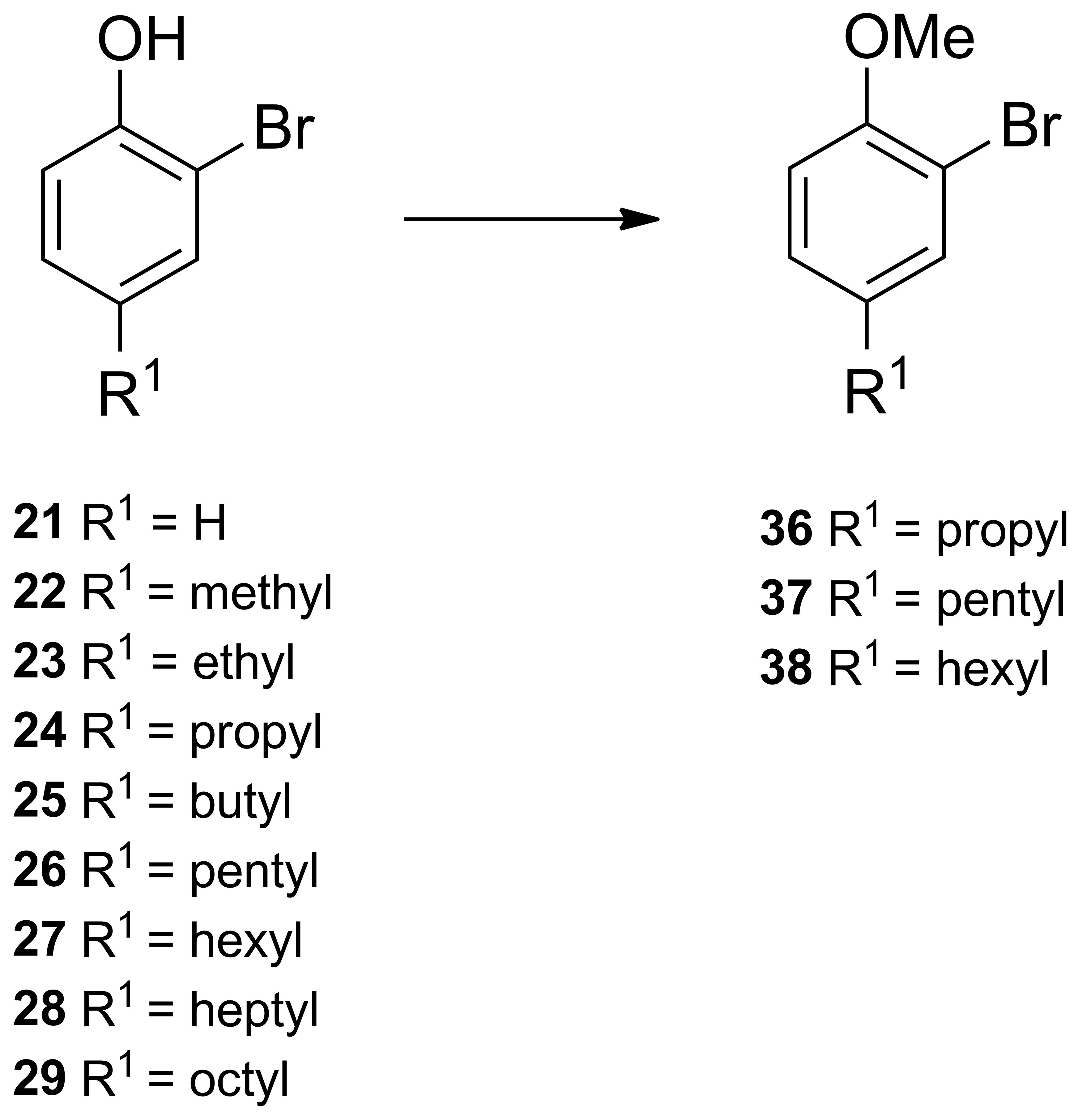

Supplement: Figure S2 — Methylation of 2-bromo-4-alkylphenols. A mixture of dichloromethane (50 mL), water (50 mL), phenol (10 mmol), sodium hydroxide (0.6 g, 15 mmol), methyl iodide (1.87 mL, 30 mmol) and benzyl tri-n-butylammonium bromide (0.36 g, l mmol) was stirred vigorously at rt for 12 h. The organic layer was then separated and the aq. layer extracted twice with dichloromethane (30 mL portions each). The combined organic extracts were evaporated under reduced pressure. The final workup of the residue was done by column chromatography (petroleum ether : ethyl acetate, 9∶ 1). (TIF) [file pone.0077739.s002.tif]

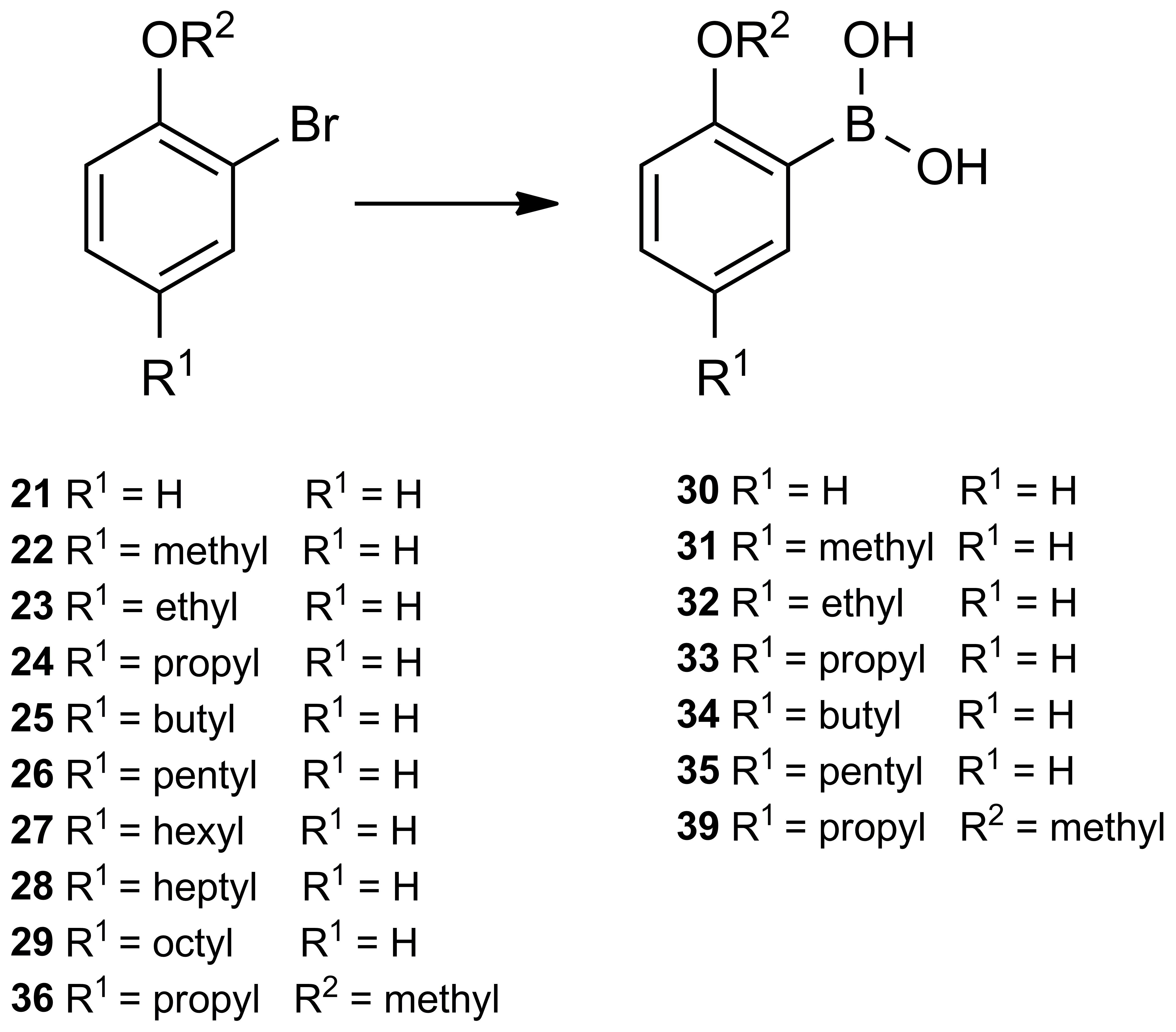

Supplement: Figure S3 — Synthesis of boronic acid derivatives. A solution of n-butyllithium (1.7 M in hexane, 38 mL) was slowly added to a cooled (–80°C) solution of 30 mmol 2-bromo-4-alkylphenol or 30 mmol of 2-bromo-1-methoxy-4-alkylphenol respectively, in dry ether (80 mL). The mixture was then allowed to warm up and stirred at rt for 2 h under an argon atmosphere. It was then cooled again (–80°C) and trimethyl borate (5.58 mL, 50 mmol) was rapidly added. The mixture was stirred at –80°C for 0.5 h and then at rt for 15 h under an argon atmosphere. Then 20 mL of 2 M aq. HCl solution were added slowly into the ice-cold reaction mixture and the mixture was stirred again for 0.5 h, while the milky white emulsion gradually became clear. The ethereal layer was then separated and the aqueous layer was extracted with diethyl ether (3 times with 100 mL each). The combined ether solutions were dried (MgSO4) and after filtration the solvent was evaporated under reduced pressure. The residual solid was recrystallized from hot diethyl ether : toluene, 3∶7) to give a white solid. (TIF) [file pone.0077739.s003.tif]

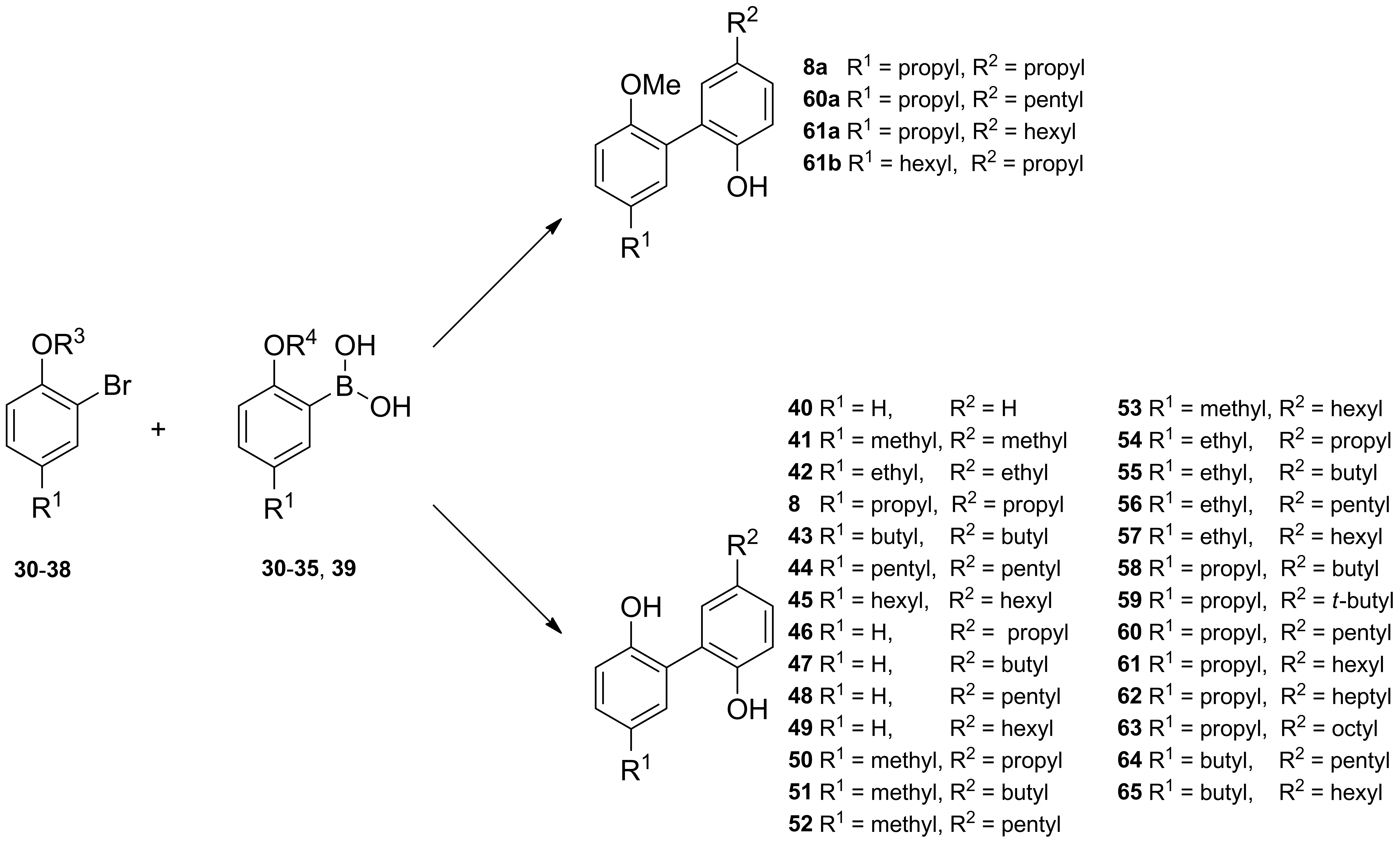

Supplement: Figure S4 — Suzuki cross-coupling. A solution of toluene (25 mL), ethanol (5 mL) and water (5 mL) in a pressure flask was flushed with argon. While keeping a positive pressure of argon 42 mmol of boronic acid, 42 mmol of 2-bromo-4-alkylphenol or 42 mmol of 2-bromo-1-methoxy-4-alkylphenol respectively, 12.3 mmol (1300 mg) of Na2CO3 and 0.108 mmol (125 mg) of tetrakis(triphenylphosphine)palladium(0) were added. The pressure flask was closed and the mixture was stirred for 18 h at 100°C. The aqueous layer was then separated and extracted three times with ethyl acetate (80 mL portions each). The combined organic extracts were evaporated under reduced pressure. The final workup of the residue was done by column chromatography (petroleum ether : ethyl acetate = 9∶ 1). (TIF) [file pone.0077739.s004.tif]

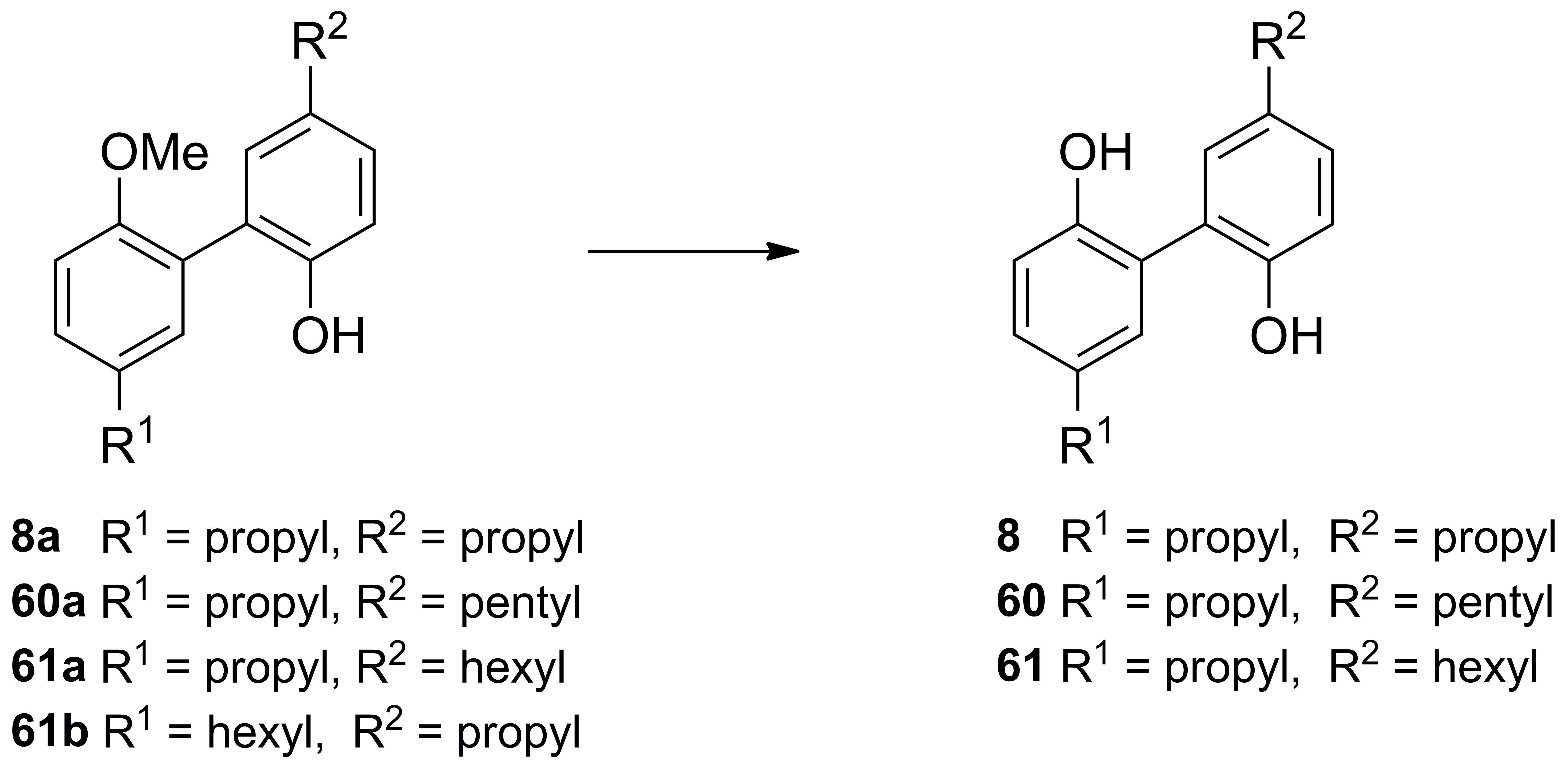

Supplement: Figure S5 — Demethylation. A solution of 14 mmol of methylated magnolol analog in dry dichloromethane (60 mL) under an argon atmosphere was cooled to −80°C. While the solution was stirred constantly, 15 mmol of BBr3 (15 ml of a 1 M solution in hexane) was added. The solution was stirred for 1.5 h at −80°C and then allowed to warm up to 0°C. Then 120 mL of water were added while the solution was at 0°C. The aqueous layer was then separated and extracted three times with dichloromethane (50 mL portions each). The combined organic extracts were evaporated under reduced pressure. The final workup of the residue was done by column chromatography (petroleum ether : ethyl acetate = 9∶ 1). (TIF) [file pone.0077739.s005.tif]

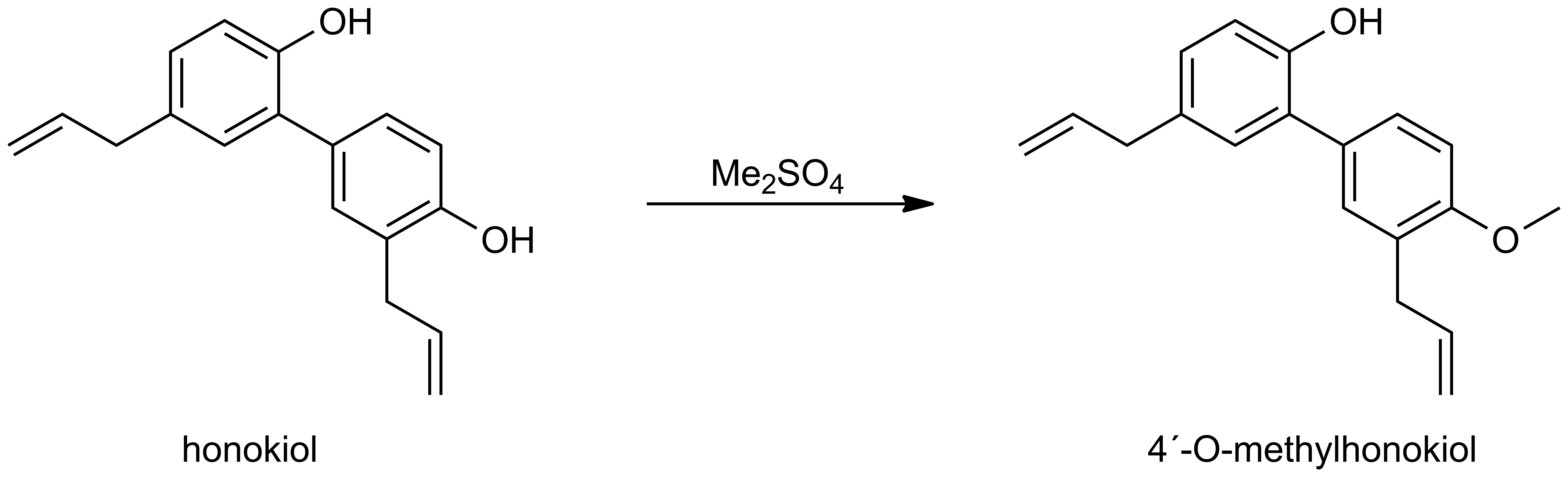

Supplement: Figure S6 — Synthesis of 4′-O-methylhonokiol. Me2SO4 (17 µL, 0.18 mmol) was added to a solution of honokiol (40 mg, 0.15 mmol) in an aqueous KOH solution (5 mL, 10%) and stirred for 1 h at 95°C. After cooling to rt HCl (1 M, 0.5 mL) was added and the mixture was subsequently extracted with chloroform (5 mL portions each). The organic layers were dried over Na2SO4 and after filtration they were concentrated under reduced pressure. The residue was subjected to HPLC separation (see below). (TIF) [file pone.0077739.s006.tif]

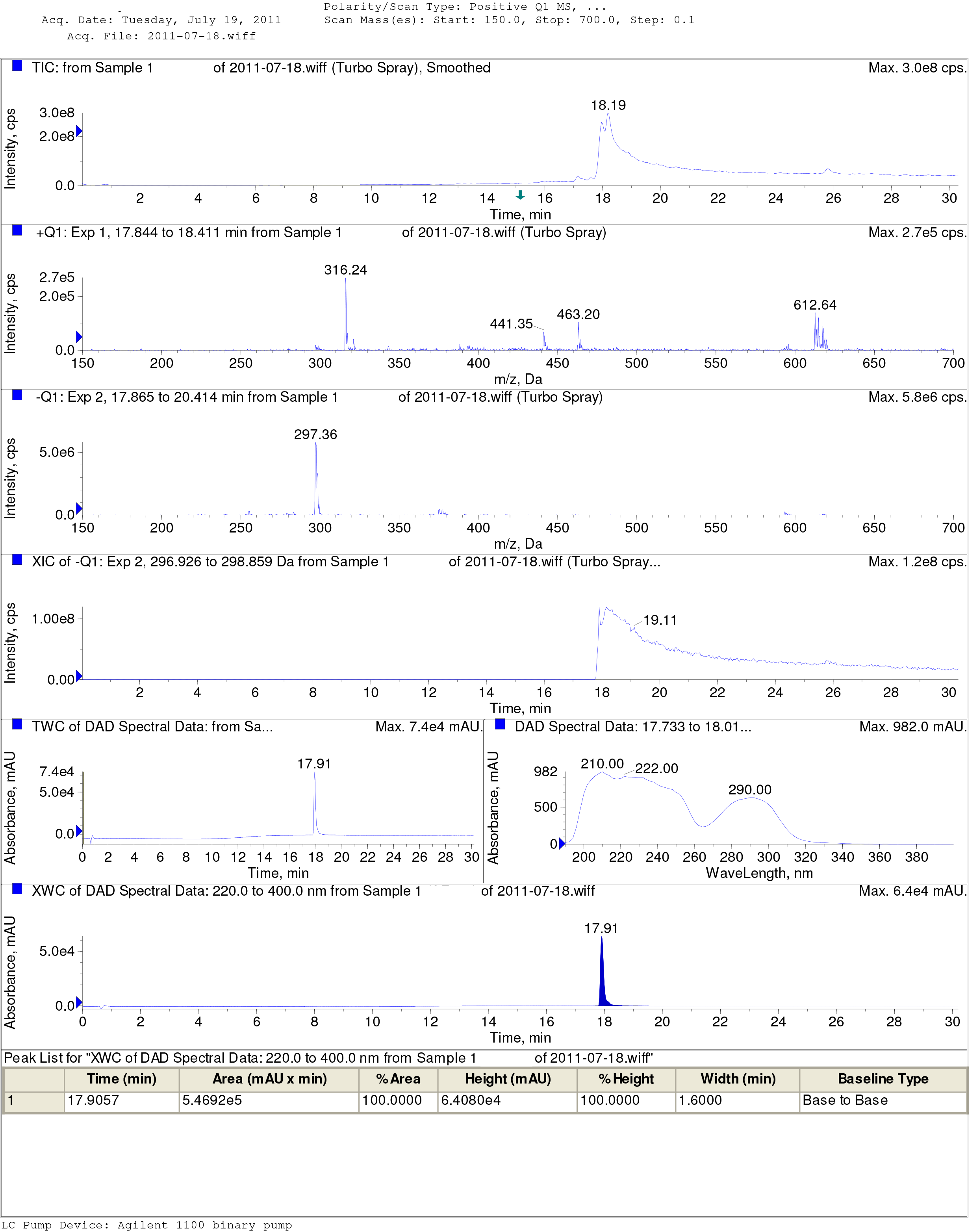

Supplement: Figure S7 — LC/ESI-MS spectrum of 60 (mass spectrum in the positive and negative mode), HPLC chromatogram (HPLC-DAD measured from 220–400 nm) of 60, and its purity determined by HPLC-DAD from 220–400 nm (100%). (TIF) [file pone.0077739.s007.tif]

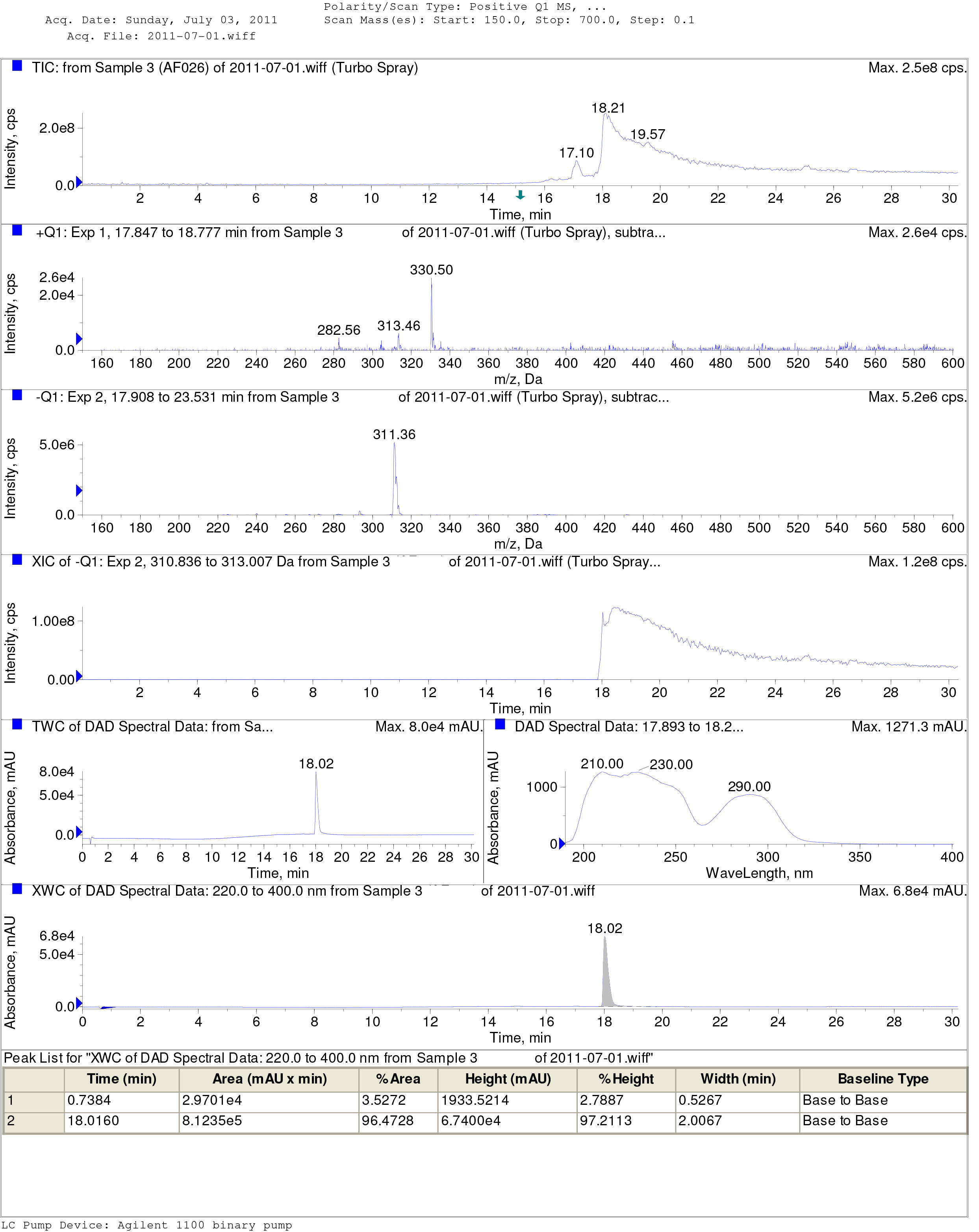

Supplement: Figure S8 — LC/ESI-MS spectrum of 61 (mass spectrum in the positive and negative mode), HPLC chromatogram (HPLC-DAD measured from 220–400 nm) of 61, and its purity determined by HPLC-DAD from 220–400 nm (100%). (TIF) [file pone.0077739.s008.tif]

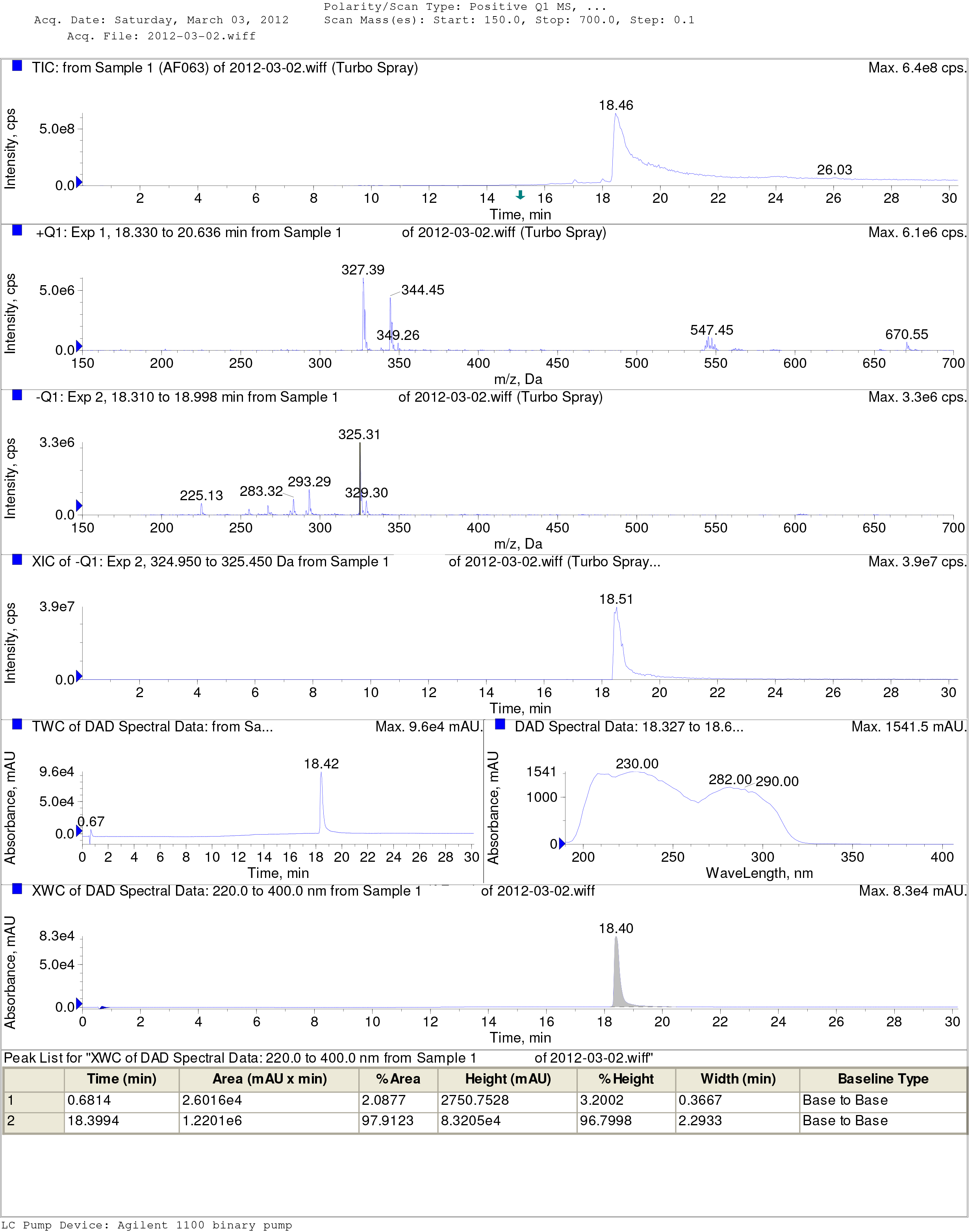

Supplement: Figure S9 — LC/ESI-MS spectrum of 61a (mass spectrum in the positive and negative mode), HPLC chromatogram (HPLC-DAD measured from 220–400 nm) of 61a, and its purity determined by HPLC-DAD from 220–400 nm (100%). (TIF) [file pone.0077739.s009.tif]

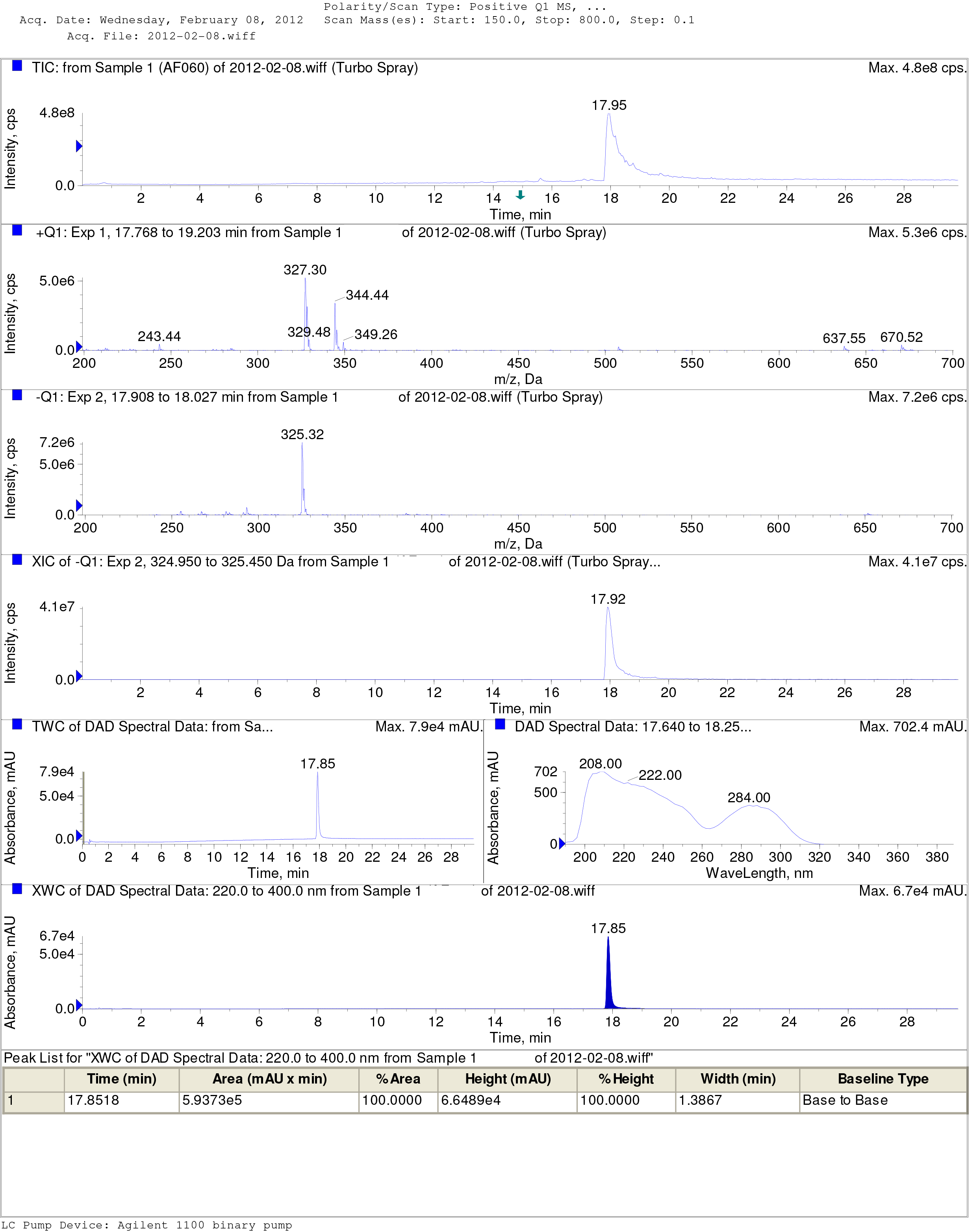

Supplement: Figure S10 — LC/ESI-MS spectrum of 61b (mass spectrum in the positive and negative mode), HPLC chromatogram (HPLC-DAD measured from 220–400 nm) of 61b, and its purity determined by HPLC-DAD from 220–400 nm (100%). (TIF) [file pone.0077739.s010.tif]
