# Supplementary material for: The Natural Product Magnolol as a Lead Structure for the Development of Potent Cannabinoid Receptor Agonists
Source: PLoS One. 2013 Oct 30;8(10):e77739. doi: 10.1371/journal.pone.0077739 (PMC3813752; doi:10.1371/journal.pone.0077739)
Supplement: Table S1 — Potencies and Efficacies of Magnolol Derivatives and Analogs at human Cannabinoid Receptor Subtypesa. aall data resulted from three independent experiments, performed in duplicates. befficacy at 10 µM compared to max. effect of the full agonist CP55,940 (1 µM) = 100%. cefficacy was determined at a concentration of 100 µM. d% inhibition of radioligand binding at 10 µM. end = not determined. (DOCX) [file pone.0077739.s011.docx]

| **Compd** | **heterologous competition vs. [³H]CP55,940** | | **cAMP accumulation assay** | | |
| --- | --- | --- | --- | --- | --- |
|  | **CB_1_** | **CB_2_** | | **CB_1_** | **CB_2_** |
|  | **K_i_ (nM)** | | | **EC_50_ (nM) / (efficacy)** | |
| **1** | 3.88 ± 0.91 | 71.6 ± 24.1 | | 6.76 ± 3.61 / (88 %)^b^ | 14.0 ± 6.8 / (34%)^b^ |
| **4** | 1.28 [21] | 1.42 [21] | | 2.28^21^ / (100%)^b^ | 1.00^21^ / (100%)^b^ |
| **9** | 3150 [34] | 1440 [34] | | 1830 / (62%)^c^ [34] | 3280 / (31%)^c^ [34] |
| **10 ** | 6460 [34] | 5610 [34] | | (4%)^b^ | (0%)^b^ |
| **11** | 8340 ± 3200 (2400) [30] | 43.3 ± 17.1 (43.9) [30] | | (42%)^b^ | (87%)^b^ |
| **12** | 2260 [34] | 416 [34] | | 9010 / (124%)^c^ [34] | 170 / (49%) [34] |
| **12a** | 267 ± 58 | 221 ± 57 | | 622 ± 284 / (112%)^b^ | 77.8 ± 20.5 / (83%)^b^ |
| **40** | > 10 (11%)^d^ | > 10 (0%)^d^ | | nd^e^ | nd^e^ |
| **41** | > 10 (23%)^d^ | > 10 (30%)^d^ | | nd^e^ | nd^e^ |
| **42** | 2130 ± 840 | 2870 ± 770 | | nd^e^ | (-3 %) |
| **43** | 2700 ± 1200 | 1590 ± 40 | | 7110 ± 1430 / (100%)^b^ | 378 ± 148 / (67%)^b^ |
| **44** | 3130 ± 1130 | 833 ± 123 | | 4540 ± 830 / (44%)^b^ | 2300 ± 710 / (62%)^b^ |
| **45** | 4640 ± 580 | 1830 ± 190 | | (-1%)^b^ | (-14%)^b^ |
| **46** | ~ 1000 (45%)^d^ | ~ 1000 (49%)^d^ | | nd^e^ | nd^e^ |
| **47** | ~ 1000 (40%)^c^ | 2030 ± 880 | | (0%)^b^ | (31%)^b^ |
| **48** | 6590 ± 2560 | 1160 ± 290 | | (50%)^b^ | (51%)^b^ |
| **49** | 6630 ± 5030 | 1500 ± 640 | | (74%)^b^ | (34%)^b^ |
| **50** | > 1000 (20%)^d^ | 7380 ± 2760 | | nd^e^ | (5 %)^b^ |
| **51** | ~ 1000 (44%)^d^ | 1690 ± 530 | | (43%)^b^ | (37%)^b^ |
| **52** | 1230 ± 470 | 517 ± 101 | | (33%)^b^ | (37%)^b^ |
| **53** | 822 ± 224 | 273 ± 96 | | (105%)^b^ | (42%)^b^ |
| **54** | ~ 1000 (43%)^d^ | 856 ± 367 | | (0%)^b^ | (12%)^b^ |
| **55** | 5760 ± 2850 | 235 ± 101 | | (88%)^b^ | (30%)^b^ |
| **56** | 634 ± 297 | 161 ± 33 | | (91%)^b^ | (91%)^b^ |
| **57** | 386 ± 29 | 83.0 ± 11.8 | | (114%)^b^ | (47%)^b^ |
| **58** | 3610 ± 170 | 468 ± 133 | | (79%)^b^ | (64%)^b^ |
| **59** | 5810 ± 2670 | 489 ± 49 | | (36%)^b^ | (36%)^b^ |
| **60** | 362 ± 113 | 37.1 ± 7.8 | | 971 ± 89 / (98%)^b^ | 258 ± 13 / (81%)^b^ |
| **60a** | 17.3 ± 1.4 | 31.0 ± 9.9 | | 37.5 ± 5.6 / (95%)^b^ | 39.9 ± 10.0 / (94%)^b^ |
| **61** | 145 ± 48 | 29.4 ± 9 | | 829 ± 278 / (102%)^b^ | 159 ± 18 / (70%)^b^ |
| **61a** | 9.57 ± 5.43 | 23.8 ± 7.1 | | 159 ± 76 / (100%)^b^ | 38.5 ± 17.1 / (100%)^b^ |
| **61b** | 313 ± 125 | 281 ± 101 | | **K_B_: 1850 ± 730 /** (0%)^b^ | 595 ± 150 / (42%)^b^ |
| **62** | 2600 ± 90 | 670 ± 28 | | (110%)^b^ | (57%)^b^ |
| **63** | 1660 ± 450 | 955 ± 418 | | (73%)^b^ | (52%)^b^ |
| **64** | 3610 ± 1350 | 234 ± 39 | | (122%)^b^ | (68%)^b^ |
| **65** | 2600 ± 90 | 670 ± 28 | | (95%)^b^ | (49%)^b^ |
