# Supplementary material for: The Natural Product Magnolol as a Lead Structure for the Development of Potent Cannabinoid Receptor Agonists
Source: PLoS One. 2013 Oct 30;8(10):e77739. doi: 10.1371/journal.pone.0077739 (PMC3813752; doi:10.1371/journal.pone.0077739)
Supplement: Table S2 — Activities of magnolol analogs and standard cannabinoid receptor ligands at human GPR18 and GPR55a. a all data result from three independent experiments, performed in duplicates. beffect of test compounds (10 µM) on β-arrestin recruitment at human GPR18 is related to the effect of Δ9-THC in a concentration of 10 µM = 100%. ceffect of test compounds (10 µM) on β-arrestin recruitment at human GPR55 is related to the effect of LPI in a concentration of 1 µM = 100%. dn.d. = not determined. (DOCX) [file pone.0077739.s012.docx]

| **Compound** | **β-arrestin recruitment assay** | | | |
| --- | --- | --- | --- | --- |
|  | **GPR18 EC_50_** (µM) | **GPR18 IC_50_** (µM) | **GPR55 EC_50_** (µM) | **GPR55 IC_50_** (µM) |
| **1** | 4.61 ± 0.50 (100)^b^ | > 10 (0)^b^ | > 10 (0)^c^ | 14.2 ± 5.4 (65)^c^ |
| **4** | > 10 (0)^b^ | 5.99 ± 1.88 (188)^b^ | > 10 (4)^c^ | 1.61 ± 0.47 (93)^c^ |
| **5** | > 10 (0)^b^ | 10.1 ± 1.3 (94)^b^ | 2.01 ± 0.66 (120)^c^ | > 10 (0)^c^ |
| **6** | > 10 (0)^b^ | 4.97 ± 1.51 (81)^b^ | > 10 (0)^c^ | 4.14 ± 1.05 (81)^c^ |
| **7** | > 10 (0)^b^ | 52 (~ 10)^b^ | > 10 (22)^c^ | 48 (~ 10)^c^ |
| **9** | > 10 (0)^b^ | > 10 (2)^b^ | > 10 (11)^c^ | > 10 (0)^c^ |
| **10** | > 10 (0)^b^ | ~ 10 (45)^b^ | > 10 (27)^c^ | ~ 10 (42)^c^ |
| **11** | > 10 (0)^b^ | > 10 (37)^b^ | > 10 (4)^c^ | 7.77 ± 0.97 (87)^c^ |
| **12** | > 10 (0)^b^ | 30.9 ± 15.8 (78)^b^ | > 10 (24)^c^ | 13.2 ± 4.0 (96)^c^ |
| **12a** | > 10 (26)^b^ | ~ 10 (41)^b^ | > 10 (0)^c^ | **4.55 ± 1.08 (82)**^c^ |
| **40** | n.d.^d^ | n.d.^d^ | n.d.^d^ | n.d.^d^ |
| **41** | > 10 (20)^b^ | > 10 (0)^b^ | > 10 (30)^c^ | > 10 (0)^c^ |
| **42** | > 10 (6)^b^ | > 10 (12)^b^ | > 10 (23)^c^ | > 10 (0)^c^ |
| **43** | > 10 (0)^b^ | 12.0 ± 1.4 (74)^b^ | > 10 (29)^c^ | > 10 (0)^c^ |
| **44** | > 10 (0)^b^ | 12.0 ± 2.7 (128)^b^ | > 10 (26)^c^ | > 10 (2)^c^ |
| **45** | > 10 (0)^b^ | 11.7 ± 2.8 (127)^b^ | > 10 (0)^c^ | > 10 (0)^c^ |
| **46** | > 10 (0)^b^ | > 10 (0)^b^ | > 10 (25)^c^ | > 10 (37)^c^ |
| **47** | > 10 (0)^b^ | > 10 (24)^b^ | > 10 (2)^c^ | > 10 (35)^c^ |
| **48** | > 10 (0)^b^ | > 10 (18)^b^ | ~ 10 (42)^c^ | > 10 (25)^c^ |
| **49** | > 10 (0)^b^ | ~ 10 (48)^b^ | > 10 (39)^c^ | > 10 (34)^c^ |
| **50** | > 10 (0)^b^ | > 10 (21)^b^ | > 10 (26)^c^ | > 10 (4)^c^ |
| **51** | > 10 (0)^b^ | ~ 10 (44)^b^ | > 10 (18)^c^ | > 10 (0)^c^ |
| **52** | > 10 (0)^b^ | 45 (~ 10)^b^ | > 10 (9)^c^ | > 10 (0)^c^ |
| **53** | > 10 (0)^b^ | 11.5 ± 1.3 (118)^b^ | > 10 (28)^c^ | > 10 (0)^c^ |
| **54** | > 10 (0)^b^ | 10.5 ± 0.9 (67)^b^ | > 10 (0)^c^ | > 10 (19)^c^ |
| **55** | > 10 (0)^b^ | ~ 10 (40)^b^ | > 10 (2)^c^ | > 10 (18)^c^ |
| **56** | > 10 (0)^b^ | 13.6 ± 0.7 (95)^b^ | > 10 (3)^c^ | > 10 (2)^c^ |
| **57** | > 10 (0)^b^ | 12.5 ± 0.8 (113)^b^ | > 10 (10)^c^ | > 10 (0)^c^ |
| **58** | > 10 (0)^b^ | 13.5 ± 1.7 (104)^b^ | > 10 (21)^c^ | ~ 10 (44)^c^ |
| **59** | > 10 (0)^b^ | ~ 10 (54)^b^ | > 10 (0)^c^ | ~ 10 (54)^c^ |
| **60** | > 10 (0)^b^ | 14.5 ± 2.8 (118)^b^ | > 10 (28)^c^ | ~ 10 (45)^c^ |
| **60a** | > 10 (22)^b^ | ~ 10 (47)^b^ | > 10 (0)^c^ | 6.93 ± 1.06 (66)^c^ |
| **61** | > 10 (0)^b^ | 10.4 ± 1.1 (139)^b^ | > 10 (32)^c^ | ~ 10 (45)^c^ |
| **61a** | > 10 (0)^b^ | > 10 (30)^b^ | > 10 (0)^c^ | ~ 10 (58)^c^ |
| **61b** | > 10 (0)^b^ | > 10 (22)^b^ | > 10 (0)^c^ | **3.25 ± 0.29 (102)**^c^ |
| **62** | > 10 (0)^b^ | 10.6 ± 0.4 (123)^b^ | > 10 (5)^c^ | > 10 (17)^c^ |
| **63** | > 10 (0)^b^ | 10.7 ± 0.7 (123)^b^ | > 10 (0)^c^ | > 10 (0)^c^ |
| **64** | > 10 (0)^b^ | 13.9 ± 0.9 (94)^b^ | > 10 (15)^c^ | > 10 (21)^c^ |
| **65** | > 10 (0)^b^ | 11.0 ± 0.7 (116)^b^ | > 10 (36)^c^ | > 10 (15)^c^ |
