# Supplementary material for: The Natural Product Magnolol as a Lead Structure for the Development of Potent Cannabinoid Receptor Agonists
Source: PLoS One. 2013 Oct 30;8(10):e77739. doi: 10.1371/journal.pone.0077739 (PMC3813752; doi:10.1371/journal.pone.0077739)
Supplement: Dataset S1 — Analytical data and yields of synthesized compounds. (DOCX) [file pone.0077739.s013.docx]

**4`-O-Methylhonokiol (11).** ^1^H NMR (500 MHz, CDCl_3_) δ 7.28 (dd, *J* = 8.3, 2.3 Hz, 1H), 7.23 (d, *J* = 2.3 Hz, 1H), 7.05 (dd, *J* = 8.2, 2.3 Hz, 1H), 7.03 (d, *J* = 2.0 Hz, 1H), 6.96 (d, *J* = 8.3 Hz, 1H), 6.90 (d, *J* = 8.2 Hz, 1H), 6.06 – 5.92 (m, 2H), 5.12 – 5.03 (m, 5H), 3.88 (s, CH_3_, 3H), 3.43 (d, *J* = 6.7 Hz, 2H), 3.35 (d, *J* = 6.7 Hz, 2H). ^13^C NMR (126 MHz, CDCl3) δ 157.04 (C_ar_), 150.82 (C_ar_), 137.80 (C_ar_), 136.49 (CH allyl), 132.15 (C_ar_), 130.48 (C_ar_), 130.19 (C_ar_), 129.78 (C_ar_), 129.01 (C_ar_), 128.75 (C_ar_), 127.88 (C_ar_), 127.82 (C_ar_), 115.83 (C_ar_), 115.51 (C_ar_), 110.97 (C_ar_), 55.54 (CH_3_), 39.40 (CH_2_), 34.27 (CH_2_). LC/ESI-MS (positive mode) m/z 281 (M+H)^+^_,_ (negative mode) m/z 279 (M-H)^-^_,_ 97.3%. Yield 20.0%.

**5,5'-Dipropylbiphenyl-2,2'-diol (12).** ^1^H NMR (500 MHz, CDCl_3_) δ 7.12 (dd, *J* = 8.2, 2.2 Hz, CHar, 2H), 7.07 (d, *J* = 2.0 Hz, CHar, 2H), 6.94 (d, *J* = 8.2 Hz, CHar, 2H), 5.51 (s, OH, 2H), 2.58 – 2.55 (t, *J* = 7.8 Hz, CH_2_, 4H), 1.70 – 1.58 (m, CH_2_ 4H), 0.95 (t, *J* = 7.3 Hz, CH_3_, 6H). ^13^C NMR (126 MHz, CDCl_3_) δ 150.98 (C_ar_-O, 2C), 135.95 (C_ar_, 2C), 131.13 (C_ar_, 2C), 129.92 (C_ar_, 2C), 123.67 (C_ar_, 2C), 116.56 (C_ar_, 2C), 37.32 (CH_2_), 24.89 (CH_2_), 13.98 (CH_3_). LC/ESI-MS (negative mode) m/z 269 (M-H)^-^ 97.6%. EA: C 79,65(79,96), H 8,411(8,20).

**2-Bromo-4-methylphenol (22).** ^1^H NMR (500 MHz, CDCl3) δ 7.27 (d, J = 1.9 Hz, 1H), 7.01 (dd, J = 8.3, 2.0 Hz, 1H), 6.91 (d, J = 8.2 Hz, 1H), 5.32 (s, OH, 1H), 2.27 (s, CH_3_, 3H). ^13^C NMR (126 MHz, CDCl_3_) δ 149.98 (C_ar_-OH), 132.08 (C_ar_), 131.40 (C_ar_), 129.76 (C_ar_), 115.70 (C_ar_), 109.80 (C_ar_), 20.17 (CH_3_). LC/ESI-MS (negative mode) m/z 185 (M-H)^-^_,_ 74.3%. Yield 80.0%.

**2-Bromo-4-ethylphenol (23).** ^1^H NMR (500 MHz, CDCl_3_) δ 7.29 (d, *J* = 2.1 Hz, H_ar_, 1H), 7.04 (dd, *J* = 8.1, 1.9 Hz, H_ar_, 1H), 6.94 (d, *J* = 8.3 Hz, H_ar_, 1H), 5.36 (s, OH, 1H), 2.57 (q, *J* = 7.6 Hz, ar-CH_2_, 2H), 1.20 (t, *J* = 7.6 Hz, CH_3_, 3H). ^13^C NMR (126 MHz, CDCl_3_) δ 150.11 (C_ar_-O), 137.95 (C_ar_), 130.97 (C_ar_), 128.61 (C_ar_), 115.80 (C_ar_), 109.90 (C_ar_), 27.70 (CH_2_), 15.64 (CH_3_). LC/ESI-MS (negative mode) m/z 201 (M-H)^-^_,_ 100%. Yield 79.2%.

**2-Bromo-4-propylphenol (24).** ^1^H NMR (500 MHz, CDCl3) δ 7.27 (d, J = 2.1 Hz, CH_ar_, 1H), 7.02 (dd, J = 8.3, 2.1 Hz, CH_ar_, 1H), 6.93 (d, J = 8.3 Hz, CH_ar_, 1H), 5.34 (s, OH, 1H), 2.50 (t, J = 7.8 Hz,ar-CH_2_, 2H), 1.60 (tq, J = 8.0, 8.0, 4.0, 4.0, 4.0 Hz, CH_2_, 2H), 0.92 (t, J = 7.3 Hz, CH_3_, 3H). ^13^C NMR (126 MHz, CDCl_3_) δ 150.10 (C_ar_-O), 136.36 (C_ar_), 131.49 (C_ar_), 129.15 (C_ar_), 115.67 (C_ar_), 109.81 (C_ar_), 36.75 (ar-CH_2_), 24.51 (CH_2_), 13.58 (CH_3_). LC/ESI-MS (negative mode) m/z 213 (M-H)^-^_,_ 99.0%. Yield 82.0%.

**2-Bromo-4-butylphenol (25).** ^1^H NMR (500 MHz, CDCl_3_) δ 7.27 (d, *J* = 2.1 Hz, H_ar_, 1H), 7.02 (dd, *J* = 8.3, 2.1 Hz, H_ar_, 1H), 6.92 (d, *J* = 8.3 Hz, H_ar_, 1H), 5.36 (s, OH, 1H), 2.52 (t, *J* = 7.8 Hz, ar-CH_2_, 2H), 1.59 – 1.51 (m, CH_2_, 2H), 1.34 (m, CH_2_, 2H), 0.92 (t, *J* = 7.4 Hz, CH_3_, 3H). ^13^C NMR (126 MHz, CDCl_3_) δ 150.07 (C_ar_-O), 136.59 (C_ar_), 131.46 (C_ar_), 129.10 (C_ar_), 115.70 (C_ar_), 109.83 (C_ar_), 34.40 (ar-CH_2_), 33.61 (CH_2_), 22.15 (CH_2_), 13.86 (CH_3_). LC/ESI-MS (negative mode) m/z 227 (M-H)^-^_,_ 95.75%. Yield 79.0%.

**2-Bromo-4-pentylphenol (26).** ^1^H NMR (500 MHz, CDCl_3_) δ 7.27 (d, *J* = 2.0 Hz, H_ar_, 1H), 7.02 (dd, *J* = 8.3, 2.0 Hz, H_ar_, 1H), 6.93 (d, *J* = 8.3 Hz, H_ar_, 1H), 5.34 (s, OH, 1H), 2.51 (t, *J* = 7.8 Hz, CH_2_, 2H), 1.57 (m, CH_2_, 2H), 1.38 – 1.26 (m, CH_2_-CH_2_, 4H), 0.89 (t, *J* = 7.0 Hz, CH_3_, 3H). ^13^C NMR (126 MHz, CDCl_3_) δ 150.09 (C_ar_-O), 136.67 (C_ar_), 131.46 (C_ar_), 129.12 (C_ar_), 115.72 (C_ar_), 109.86 (C_ar_), 34.71 (ar-CH_2_), 31.31 (CH_2_), 31.17 (CH_2_), 22.48 (CH_2_), 13.99 (CH_3_). LC/ESI-MS (negative mode) m/z 241 (M-H)^-^_,_ 97.49%. Yield 75.0%.

**2-Bromo-4-hexylphenol (27).** ^1^H NMR (500 MHz, CDCl_3_) δ 7.27 (d, *J* = 2.0 Hz, H_ar_, 1H), 7.02 (dd, *J* = 8.3, 1.7 Hz, H_ar_, 1H), 6.93 (d, *J* = 8.3 Hz, H_ar_, 1H), 5.35 (s, OH, 1H), 2.51 (t, *J* = 7.8 Hz, ar-CH_2_, 2H), 1.60 – 1.53 (m, CH_2_, 2H), 1.34 – 1.26 (m, CH_2_-CH_2_-CH_2_, 6H), 0.89 (t, *J* = 6.8 Hz, CH_3_, 3H). ^13^C NMR (126 MHz, DMSO) δ 150.08 (C_ar_-O), 136.66 (C_ar_), 131.45 (C_ar_), 129.11 (C_ar_), 115.71 (C_ar_), 109.85 (C_ar_), 34.73 (CH_2_), 31.64 (CH_2_), 31.46 (CH_2_), 28.79 (CH_2_), 22.56 (CH_2_), 14.05 (CH_3_). LC/ESI-MS (negative mode) m/z 255 (M-H)^-^_,_ 96.5%. Yield 76.0%.

**2-Bromo-4-heptylphenol (28).** ^1^H NMR (500 MHz, CDCl3) δ 7.27 (d, J = 2.0 Hz, H_ar_, 1H), 7.02 (dd, J = 8.3, 2.0 Hz, H_ar_, 1H), 6.92 (d, J = 8.3 Hz, H_ar_, 1H), 5.33 (s, OH, 1H), 2.51 (t, J = 8.0 Hz, ar-CH_2_, 2H), 1.34 – 1.23 (m, CH_2_-CH_2_-CH_2_-CH_2_-CH_2_, 10H), 0.88 (t, J = 7.1 Hz, CH_3_, 3H). ^13^C NMR (126 MHz, CDCl_3_) δ 150.09 (C_ar_-O), 136.68 (C_ar_), 131.46 (C_ar_), 129.12 (C_ar_), 115.72 (C_ar_), 109.86 (C_ar_), 34.75 (ar-CH_2_), 31.78 (CH_2_), 31.51 (CH_2_), 29.11 (CH_2_), 29.10 (CH_2_), 22.64 (CH_2_), 14.07 (CH_3_). LC/ESI-MS: (negative mode) m/z 269 (M-H)^-^_,_ 96.0%. Yield 78.0%.

**2-Bromo-4-octylphenol (29).** ^1^H NMR (500 MHz, CDCl_3_) δ 7.27 (d, *J* = 2.0 Hz, H_ar_, 1H), 7.02 (dd, *J* = 8.3, 2.0 Hz, H_ar_, 1H), 6.92 (d, *J* = 8.3 Hz, H_ar_, 1H), 5.33 (s, OH 1H), 2.51 (t, *J* = 6.0 Hz, ar-CH_2_, 2H), 1.60 – 1.52 (m, CH_2_, 2H), 1.34 – 1.22 (m, CH_2_-CH_2_-CH_2_-CH_2_-CH_2_, 10H), 0.88 (t, *J* = 7.0 Hz, CH_3_, 3H). ^13^C NMR (126 MHz, CDCl_3_) δ 150.09 (C_ar_-O), 136.68 (C_ar_), 131.46 (C_ar_), 129.13 (C_ar_), 115.72 (C_ar_), 109.87 (C_ar_), 34.75 (CH_2_), 31.86 (CH_2_), 31.51 (CH_2_), 29.41 (CH_2_), 29.22 (CH_2_), 29.14 (CH_2_), 22.65 (CH_2_), 14.09 (CH_3_). LC/ESI-MS: (negative mode) m/z 285 (M-H)^-^_,_ 94.7%. Yield 80.0%.

**2-Hydroxy-5-methylphenylboronic acid (31).** Identity confirmed by LC/ESI-MS

**5-Ethyl-2-hydroxyphenylboronic acid (32).** Identity confirmed by LC/ESI-MS

**2-Hydroxy-5-propylphenylboronic acid (33).** Identity confirmed by LC/ESI-MS

**5-Butyl-2-hydroxyphenylboronic acid (34).** Identity confirmed by LC/ESI-MS

**2-Hydroxy-5-pentylphenylboronic acid (35).** Identity confirmed by LC/ESI-MS

**2-Bromo-1-methoxy-4-propylbenzene (36).** ^1^H NMR (500 MHz, CDCl_3_) δ 7.36 (d, *J* = 1.8 Hz, CH_ar_, 1H), 7.06 (dd, *J* = 8.3, 1.6 Hz, CH_ar_, 1H), 6.81 (d, *J* = 8.3 Hz, CH_ar_, 1H), 3.87 (s, O-CH_3_ 3H), 2.50 (t, *J* = 7.5 Hz, ar-CH_2_, 2H), 1.60 (qt, J = 8.0, 8.0, 8.0, 4.0, 4.0 Hz, CH_2_, 2H), 0.92 (t, J = 7.3 Hz, CH_3_, 3H). ^13^C NMR (126 MHz, CDCl3) δ 153.87 (C_ar_-O), 136.38 (C_ar_), 133.15 (C_ar_), 128.28 (C_ar_), 111.81 (C_ar_), 111.29 (C_ar_), 56.25 (O-CH_3_), 36.73 (CH_2_), 24.53 (CH_2_), 13.63 (CH_3_). LC/ESI-MS positive mode m/z 149 (M+H-Br)^+^_,_ 97.8%. Yield 89%.

**2-Bromo-1-methoxy-4-pentylbenzene (37).** ^1^H NMR (500 MHz, CDCl_3_) δ 7.36 (d, *J* = 2.2 Hz, H_ar_, 1H), 7.06 (dd, *J* = 8.3, 2.2 Hz, H_ar_, 1H), 6.81 (d, *J* = 8.3 Hz, H_ar_, 1H), 3.87 (s, O-CH_3_, 3H), 2.52 (t, *J* = 7.5 Hz, ar-CH_2_, 2H), 1.57 (m, CH_2_, 2H), 1.37 – 1.26 (m, CH_2_-CH_2_, 4H), 0.89 (t, *J* = 7.1 Hz, CH_3_, 3H). ^13^C NMR (126 MHz, CDCl_3_) δ 153.84 (C_ar_-O), 136.65 (C_ar_), 133.10 (C_ar_), 128.21 (C_ar_), 111.82 (C_ar_), 111.31 (C_ar_), 56.26 (O-CH_3_), 34.65 (CH_2_), 31.33 (CH_2_), 31.16 (CH_2_), 22.48 (CH_2_), 13.99 (CH_3_). LC/ESI-MS (negative mode) m/z 255 (M-H)^-^_,_ 97.8%. Yield 91 %.

**2-Bromo-4-hexyl-1-methoxybenzene (38).** ^1^H NMR (500 MHz, CDCl_3_) δ 7.36 (d, *J* = 2.2 Hz, H_ar_, 1H), 7.06 (dd, *J* = 8.3, 2.2 Hz, H_ar_, 1H), 6.81 (d, *J* = 8.3 Hz, H_ar_, 1H), 3.87 (s, *J* = 2.7 Hz, O-CH_3_, 3H), 2.52 (t, *J* = 8.0 Hz, ar-CH_2_, 2H), 1.60 – 1.52 (m, CH_2_, 2H), 1.35 – 1.26 (m, CH_2_-CH_2_-CH_2_, 6H), 0.88 (t, *J* = 6.0 Hz, CH_3_, 3H). ^13^C NMR (126 MHz, CDCl_3_) δ 153.84 (C_ar_-O), 136.66 (C_ar_), 133.10 (C_ar_), 128.20 (C_ar_), 111.82 (C_ar_), 111.31 (C_ar_), 77.25 (C_ar_), 77.00 (C_ar_), 76.74 (C_ar_), 56.26 (O-CH_3_), 34.69 (CH_2_), 31.66 (CH_2_), 31.45 (CH_2_), 28.82 (CH_2_), 22.57 (CH_2_), 14.06 (CH_3_). LC/ESI-MS: (negative mode) m/z 269 (M-H)^-^_,_ 98.8%. Yield 91 %

**2-Methoxy-5-propylphenylboronic acid (39).** ^1^H NMR (500 MHz, DMSO) δ 7.61 (s, B-OH, 2H), 7.38 (d, *J* = 2.4 Hz, Ch_ar_, 1H), 7.17 (dd, *J* = 8.4, 2.4 Hz, Ch_ar_, 1H), 6.87 (d, *J* = 8.4 Hz, Ch_ar_, 1H), 3.77 (s, O-CH_3_, 3H), 2.47 (t, *J* = 7.5 Hz, ar-CH_2_, 2H), 1.53 (tq, *J* = 8.0, 8.0, 4.0, 4.0, 4.0 Hz, CH_2_, 2H), 0.86 (t, *J* = 7.3 Hz, CH_3_, 3H). ^13^C NMR (126 MHz, DMSO) δ 161.93 (C_ar_-O), 135.49 (C_ar_), 133.6 (C_ar_), 131.46 (C_ar_), 121.22 (C_ar_), 110.34 (C_ar_), 55.47 (O-CH_3_), 36.54 (ar-CH_2_), 24.44 (CH_2_), 13.70 (CH_3_). LC/ESI-MS: (positive mode) m/z 195 (M+H)^+^_,_ (negative mode) m/z 193 (M-H)^-^, 100%. Yield 51%.

**5,5'-Dimethylbiphenyl-2,2'-diol (41)** ^1^H NMR (500 MHz, CDCl_3_) δ 7.10 (dd, *J* = 8.2, 1.6 Hz, CH_ar_, 2H), 7.07 (d, *J* = 2.1 Hz, CH_ar_, 2H), 6.91 (d, *J* = 8.2 Hz, CH_ar_, 2H), 5.53 (s, OH, 2H), 2.33 (s, CH_3_, 6H). ^13^C NMR (126 MHz, CDCl_3_) δ 150.54 (C_ar_-O, 2C), 131.54 (C_ar_, 2C), 130.83 (C_ar_, 2C), 130.09 (C_ar_, 2C), 123.57 (C_ar_, 2C), 116.43 (C_ar_, 2C), 20.46 (CH_3_). Mp. 156 °C. LC/ESI-MS (negative mode) m/z 213 (M-H)^-^_,_ 100%. Yield 31.0%.

**5,5'-Diethylbiphenyl-2,2'-diol (42)** ^1^H NMR (500 MHz, CDCl_3_) δ 7.15 (dd, *J* = 8.3, 2.3 Hz, CH_ar_, 2H), 7.10 (d, *J* = 2.1 Hz, CH_ar_, 2H), 6.95 (d, *J* = 8.3 Hz, CH_ar_, 2H), 5.46 (s, OH, 2H), 2.64 (q, *J* = 7.6 Hz, CH_2_, 4H), 1.24 (t, *J* = 7.6 Hz, CH_3_, 6H). ^13^C NMR (126 MHz, CDCl_3_) δ 150.72 (C_ar_, 2C), 137.24 (C_ar_, 2C), 129.66 (C_ar_, 2C), 128.97 (C_ar_, 2C), 123.61 (C_ar_, 2C), 116.42 (C_ar_, 2C), 27.91 (CH_2_), 15.70 (CH_3_)_._ Mp. 126-127 °C. LC/ESI-MS (negative mode) m/z 241 (M-H)^-^_,_ 95.4%. Yield 35.0%.

**5,5'-Dipropylbiphenyl-2,2'-diol (12)** ^1^H NMR (500 MHz, CDCl_3_) δ 7.12 (dd, *J* = 8.2, 2.2 Hz, CHar, 2H), 7.07 (d, *J* = 2.0 Hz, CHar, 2H), 6.94 (d, *J* = 8.2 Hz, CHar, 2H), 5.51 (s, OH, 2H), 2.58 – 2.55 (t, *J* = 7.8 Hz, CH_2_, 4H), 1.70 – 1.58 (m, CH_2_ 4H), 0.95 (t, *J* = 7.3 Hz, CH_3_, 6H). ^13^C NMR (126 MHz, CDCl_3_) δ 150.98 (C_ar_-O, 2C), 135.95 (C_ar_, 2C), 131.13 (C_ar_, 2C), 129.92 (C_ar_, 2C), 123.67 (C_ar_, 2C), 116.56 (C_ar_, 2C), 37.32 (CH_2_), 24.89 (CH_2_), 13.98 (CH_3_). Mp. 142-143 °C. LC/ESI-MS (negative mode) m/z 269 (M-H)^-^_,_ 97.6%. Yield 29.8%.

**5,5'-Dibutylbiphenyl-2,2'-diol (43)** ^1^H NMR (500 MHz, CDCl_3_) δ 7.12 (dd, *J* = 8.2, 2.2 Hz, CH_ar_, 2H), 7.07 (d, *J* = 2.1 Hz, CH_ar_, 2H), 6.94 (d, *J* = 8.2 Hz, CH_ar_, 2H), 5.42 (s, OH, 2H), 2.59 (t, *J* = 7.8 Hz, ar-CH_2_, 4H), 1.64 – 1.56 (m, CH_2_, 4H), 1.41 – 1.33 (m, CH_2_, 4H), 0.93 (t, *J* = 7.4 Hz, CH_3_, 6H). ^13^C NMR (126 MHz, CDCl_3_) δ 150.77 ((C_ar_-O), 2C), 136.00 ((C_ar_), 2C), 130.87 ((C_ar_), 2C), 129.74 ((C_ar_), 2C), 123.40 ((C_ar_), 2C), 116.39 ((C_ar_), 2C), 34.74 (ar-CH_2_), 33.81 (CH_2_), 22.34 (CH_3_), 13.93 (CH_3_). Mp. 78-79 °C. LC/ESI-MS (negative mode) m/z 297 (M-H)^-^_,_ 100%. Yield 34.2%.

**5,5'-Dipentylbiphenyl-2,2'-diol (44)** ^1^H NMR (500 MHz, CDCl_3_) δ 7.12 (dd, *J* = 8.3, 2.2 Hz, Ch_ar_, 2H), 7.07 (d, *J* = 2.2 Hz, Ch_ar_, 2H), 6.94 (d, *J* = 8.2 Hz, Ch_ar_, 2H), 5.44 (s, OH, 2H), 2.61 – 2.55 (t, *J* = 7.8 Hz, ar-CH_2_, 4H), 1.67 – 1.55 (m, 4H, CH_2_, 4H), 1.40 – 1.28 (m, CH_2_-CH_2_, 8H), 0.90 (t, *J* = 7.0 Hz, CH_3_, 6H). ^13^C NMR (126 MHz, CDCl_3_) δ 150.74 (C_ar_-O, 2C), 136.06 (C_ar_, 2C), 130.87 (C_ar_, 2C), 129.73 (C_ar_, 2C), 123.44 (C_ar_, 2C), 116.40 (C_ar_, 2C), 35,02 (ar-CH_2_, 2C), 31.49 (CH_2_, 2C), 31.33 (CH_2_, 2C), 22.51 (CH_2_, 2C), 14.02 (CH_3_, 2C). Mp. 53-54 °C. LC/ESI-MS (negative mode) m/z 325 (M-H)^-^_,_ 99.3%. Yield 30.9%.

**5,5'-Dihexylbiphenyl-2,2'-diol (45)** ^1^H NMR (500 MHz, CDCl3) δ 7.12 (dd, *J* = 8.2, 2.2 Hz, CH_ar_, 2H), 7.07 (d, *J* = 2.2 Hz, CH_ar_, 2H), 6.94 (d, *J* = 8.2 Hz, CH_ar_, 2H), 5.22 (s, OH 2H), 2.58 (t, *J* = 8.0 Hz, ar-CH_2_, 4H), 1.65 – 1.57 (m, CH_2_, 4H), 1.39 – 1.27 (m, CH_2_-CH_2_-CH_2_, 12H), 0.92 – 0.86 (t, *J* = 7.0 Hz, CH_3_, 6H) ^13^C NMR (126 MHz, CDCl3) δ 150.74 (C_ar_-O, 2C), 136.07 (C_ar_, 2C), 130.89 (C_ar_, 2C), 129.71 (C_ar_, 2C), 123.49 (C_ar_, 2C), 116.41 (C_ar_, 2C), 35.07 (ar-CH_2_, 2C), 31.70 (CH_2_, 2C), 31.62 (CH_2_, 2C), 28.97 (CH_2_, 2C), 22.60 (CH_2_, 2C), 14.07 (CH_3_, 2C). Mp. 49-50°C. LC/ESI-MS (negative mode) m/z 353 (M-H)^-^_,_ 99.3%. Yield 33.1%.

**5-Propylbiphenyl-2,2'-diol (46)** ^1^H NMR (500 MHz, CDCl_3_) δ 7.34 – 6.93 (m, CH_ar_, 7H), 5.67 (s, OH, 1H), 5.54 (s, OH, 1H), 2.57 (t, *J* = 7.5 Hz, ar-CH_2_, 2H), 1.68 – 1.60 (m, CH_2_, 2H), 0.95 (t, *J* = 7.3 Hz, CH_3_, 3H). ^13^C NMR (126 MHz, CDCl_3_) δ 152.92 (C_ar_-O), 150.71 (C_ar_-O), 135.92 (C_ar_), 131.24 (C_ar_), 131.08 (C_ar_), 129.90 (C_ar_), 129.82 (C_ar_), 123.93 (C_ar_), 123.23 (C_ar_), 121.51 (C_ar_), 116.64 (C_ar_), 116.42 (C_ar_), 37.12 (ar-CH_2_), 24.69 (CH_2_), 13.78 (CH_3_). LC/ESI-MS (negative mode) m/z 227 (M-H)^-^_,_ 98.7%. Yield 28.7%.

**5-Butylbiphenyl-2,2'-diol (47)** ^1^H NMR (500 MHz, CDCl_3_) δ 7.33 – 6.92 (m, Ch_ar_, 7H), 5.77 (s, OH, 2H), 2.59 (t, *J* = 7.8 Hz, ar-CH_2_, 2H), 1.63 – 1.56 (m, CH_2_, 2H), 1.37 (m, CH_2_, 2H), 0.93 (t, *J* = 7.4 Hz, CH_3_, 3H). ^13^C NMR (126 MHz, CDCl_3_) δ 152.91 (C_ar_-O), 150.65 (C_ar_-O), 136.09 (C_ar_), 131.25 (C_ar_), 131.03 (C_ar_), 129.77 (C_ar_), 129.74 (C_ar_), 124.08 (C_ar_), 123.39 (C_ar_), 121.47 (C_ar_), 116.63 (C_ar_), 116.42 (C_ar_), 34.71 (ar-CH_2_), 33.78 (CH_2_), 22.29 (CH_2_), 13.91 (CH_3_). LC/ESI-MS (negative mode) m/z 241 (M-H)^-^_,_ 98.3%. Yield 30.2%.

**5-Pentylbiphenyl-2,2'-diol (48)** ^1^H NMR (500 MHz, CDCl_3_) δ 7.30 – 6.91 (m, CH_ar_, 7H), 5.56 (s, OH, 2H), 2.58 (t, *J* = 8.0 Hz, ar-CH_2_, 2H), 1.61 (m, *J* = 15.2, 7.5 Hz, CH_2_, 2H), 1.37 – 1.30 (m, CH_2_-CH_2,_ 4H), 0.90 (t, *J* = 7.1 Hz, CH_3_, 3H). ^13^C NMR (126 MHz, CDCl_3_) δ 152.87 (C_ar_-O), 150.62 (C_ar_-O), 136.16 (C_ar_), 131.29 (C_ar_), 131.06 (C_ar_), 129.74 (C_ar_), 129.73 (C_ar_), 124.20 (C_ar_), 123.51 (C_ar_), 121.50 (C_ar_), 116.66 (C_ar_), 116.44 (C_ar_), 35.01 (ar-CH_2_), 31.47 (CH_2_), 31.32 (CH_2_), 22.50 (CH_2_), 14.01 (CH_3_). LC/ESI-MS (negative mode) m/z 255 (M-H)^-^_,_ 98.6%. Yield 29.3%.

**5-Hexylbiphenyl-2,2'-diol (49)** ^1^H NMR (500 MHz, CDCl_3_) δ 7.40 – 6.87 (m, Ch_ar_, 7H), 5.50 (s, OH, 2H), 2.58 (t, *J* = 7.5 Hz, ar-CH_2_, 2H), 1.61 (m,CH_2_, 2H), 1.38 – 1.28 (m, CH_2_-CH_2_-CH_2_, 6H), 0.89 (t, *J* = 2.0 Hz, CH_3_, 3H). ^13^C NMR (126 MHz, CDCl_3_) δ 152.90, 150.64, 136.18, 131.26, 131.02, 129.78 (2C), 124.05, 123.35, 121.50, 116.64, 116.43, 35.05, 31.69, 31.61, 28.95, 22.58, 14.06. ^13^C NMR (126 MHz, CDCl_3_) δ 152.90 (C_ar_-O), 150.64 (C_ar_-O), 136.18 (C_ar_), 131.26 (C_ar_), 131.02 (C_ar_), 129.78 (C_ar_, 2C), 124.05 (C_ar_), 123.35 (C_ar_), 121.50 (C_ar_), 116.64 (C_ar_), 116.43 (C_ar_), 35.05 (ar-CH_2_,), 31.69 (CH_2_,), 31.61 (CH_2_,), 28.95 (CH_2_,), 22.58 (CH_2_,) 14.06 (CH_3_). LC/ESI-MS (negative mode) m/z 269 (M-H)^-^_,_ 100%. Yield 28.1%.

**5-Methyl-5'-propylbiphenyl-2,2'-diol (50)** ^1^H NMR (500 MHz, CDCl_3_) δ 7.13 – 7.06 (m, CH_ar_, 4H), 6.93 (dd, *J* = 8.2, 5.3 Hz, CH_ar_, 2H), 5.59 (s, OH, 2H), 2.56 (t, *J* = 7.5 Hz, ar-CH_2_ ,2H), 2.33 (s, ar-CH_3,_ 3H), 1.68 – 1.60 (m, CH_2_ ,2H), 0.95 (t, *J* = 7.3 Hz, CH_3_, 3H). ^13^C NMR (126 MHz, CDCl_3_) δ 150.72 (C_ar_-O), 150.59 (C_ar_-O), 135.82 (C_ar_), 131.55 (C_ar_), 130.99 (C_ar_), 130.81 (C_ar_), 130.33 (C_ar_), 129.75 (C_ar_), 123.70 (C_ar_), 123.49 (C_ar_), 116.46 (C_ar_), 116.38 (C_ar_), 37.14 (ar-CH_2_), 24.70 (CH_2_), 20.48 (CH_3_), 13.80 (CH_3_). LC/ESI-MS (negative mode) m/z 241 (M-H)^-^_,_ 100%. Yield 27.5%.

**5-Butyl-5'-methylbiphenyl-2,2'-diol (51)** ^1^H NMR (500 MHz, CDCl_3_) δ 7.13 – 7.06 (m, 4H), 6.93 (dd, *J* = 8.2, 3.9 Hz, 2H), 5.55 (s, 2H), 2.59 (t, *J* = 7.8 Hz, ar-CH_2_, 2H), 2.33 (s, CH_3_, 3H), 1.63 – 1.56 (m, CH_2_, 2H), 1.37 (m, CH_2_, 2H), 0.93 (t, *J* = 7.4 Hz, CH_3_, 3H). ^13^C NMR (126 MHz, CDCl_3_) δ 150.71 (C_ar_-O), 150.62 (C_ar_-O), 136.03 (C_ar_), 131.53 (C_ar_), 130.92 (C_ar_), 130.79 (C_ar_), 130.34 (C_ar_), 129.72 (C_ar_), 123.66 (C_ar_), 123.46 (C_ar_), 116.46 (C_ar_), 116.39 (C_ar_), 34.74 (ar-CH_2_), 33.81 (CH_2_), 22.33 (CH_2_), 20.48 (CH_3_), 13.93 (CH_3_). mp 86-87 °C. LC/ESI-MS (negative mode) m/z 255 (M-H)^-^_,_ 100%. Yield 27.9%.

**5-Methyl-5'-pentylbiphenyl-2,2'-diol (52)** ^1^H NMR (500 MHz, CDCl_3_) δ 7.10 – 7.05 (m, Ch_ar_, 4H), 6.90 (dd, *J* = 8.2, 3.9 Hz, CH_ar_, 2H), 5.72 (s, OH, 2H), 2.56 (t, *J* = 7.8 Hz, CH_2_, 2H), 2.31 (s, CH_3_, 3H), 1.63 – 1.55 (m, CH_2_, 2H), 1.35 – 1.28 (m, CH_2_-CH_2_ 4H), 0.88 (t, *J* = 7.1 Hz, CH_3_, 3H). ^13^C NMR (126 MHz, CDCl_3_) δ 150.67 (C_ar_-O), 150.58 (C_ar_-O), 136.03 (C_ar_), 131.56 (C_ar_), 130.93 (C_ar_), 130.75 (C_ar_), 130.25 (C_ar_), 129.62 (C_ar_), 123.83 (C_ar_), 123.63 (C_ar_), 116.46 (C_ar_), 116.38 (C_ar_), 35.01 (ar-CH_2_), 31.47 (CH_2_), 31.32 (CH_2_), 22.49 (CH_2_), 20.46 (CH_3_), 14.00 (CH_3_). LC/ESI-MS (negative mode) m/z 269 (M-H)^-^_,_ 93.3%. Yield 29.3%.

**5-Hexyl-5'-methylbiphenyl-2,2'-diol (53)** ^1^H NMR (500 MHz, CDCl_3_) δ 7.13 – 7.07 (m, CH_ar_, 4H), 6.92 (dd, *J* = 8.2, 3.6 Hz, CH_ar_, 2H), 5.63 (s, OH, 2H), 2.58 (t, *J* = 7.8 Hz, CH_2_, 2H), 2.33 (s, CH_3_, 3H), 1.64 – 1.57 (m, CH_2_, 2H), 1.38 – 1.28 (m, CH_2_-CH_2_-CH_2_, 6H), 0.89 (t, *J* = 7.0 Hz, CH_3_, 3H). ^13^C NMR (126 MHz, CDCl_3_) δ 150.68 (C_ar_-O), 150.60 (C_ar_-O), 136.08 (C_ar_), 131.56 (C_ar_), 130.93 (C_ar_), 130.79 (C_ar_), 130.31 (C_ar_), 129.68 (C_ar_), 123.75 (C_ar_), 123.54 (C_ar_), 116.47 (C_ar_), 116.39 (C_ar_), 35.06 (ar-CH_2_), 31.69 (CH_2_), 31.63 (CH_2_), 28.96 (CH_2_), 22.59 (CH_2_), 20.48 (CH_3_), 14.07 (CH_3_). LC/ESI-MS (negative mode) m/z 283 (M-H)^-^_,_ 100%. Yield 26.4%.

**5-Ethyl-5'-propylbiphenyl-2,2'-diol (54)** ^1^H NMR (500 MHz, CDCl_3_) δ 7.16 – 7.07 (m, CH_ar_, 4H), 6.94 (dd, *J* = 8.2, 4.3 Hz,CH_ar_, 2H), 5.57 (s, OH, 2H), 2.64 (q, *J* = 7.6 Hz, CH_2_, 2H), 2.57 (t, *J* = 7.8 Hz, CH_2_, 2H), 1.68 – 1.60 (m, CH_2_, 2H), 1.24 (t, *J* = 7.6 Hz, CH_3_, 3H), 0.96 (t, *J* = 7.3 Hz, CH_3_, 3H). ^13^C NMR (126 MHz, CDCl_3_) δ 150.73 (C_ar_-O, 2C), 137.33 (C_ar_), 135.80 (C_ar_), 130.98 (C_ar_), 130.38 (C_ar_), 129.74 (C_ar_), 129.15 (C_ar_), 123.64 (C_ar_), 123.55 (C_ar_), 116.47 (C_ar_), 116.38 (C_ar_), 37.13 (ar-CH_2_), 27.96 (ar-CH_2_), 24.70 (CH_2_), 15.76 (CH_3_), 13.79 (CH_3_). Mp. 122-123 °C. LC/ESI-MS (negative mode) m/z 255 (M-H)^-^_,_ 97.0%. Yield 29.4%.

**5-Butyl-5'-ethylbiphenyl-2,2'-diol (55)** ^1^H NMR (500 MHz, CDCl_3_) δ 7.16 – 7.07 (m, CH_ar_, 4H), 6.95 (dd, *J* = 8.2, 5.7 Hz, CH_ar_, 2H), 5.42 (s, OH, 2H), 2.64 (q, *J* = 7.6 Hz, CH_2_, 2H), 2.59 (t, *J* = 7.8 Hz, CH_2_, 2H), 1.64 – 1.56 (m, CH_2_, 2H), 1.37 (m, CH_2_, 2H), 1.24 (t, *J* = 7.6 Hz, CH_3_, 3H), 0.93 (t, *J* = 7.4 Hz, CH_3_, 3H). ^13^C NMR (126 MHz, CDCl_3_) δ 150.81 (C_ar_-O), 150.77 (C_ar_-O), 137.34 (C_ar_), 136.03 (C_ar_), 130.90 (C_ar_), 130.37 (C_ar_), 129.75 (C_ar_), 129.22 (C_ar_), 123.54 (C_ar_), 123.46 (C_ar_), 116.49 (C_ar_), 116.40 (C_ar_), 34.75 (ar-CH_2_), 33.83 (ar-CH_2_), 27.99 (CH_2_), 22.35 (CH_2_), 15.78 (CH_3_), 13.94 (CH_3_). LC/ESI-MS (positive mode) m/z 271 (M+H)^+^_,_ (negative mode) m/z 269 (M-H)^-^, 100%. Yield 26.1%.

**5-Ethyl-5'-pentylbiphenyl-2,2'-diol (56)** ^1^H NMR (500 MHz, CDCl_3_) δ 7.15 – 7.07 (m, CH_ar_, 4H), 6.94 (dd, *J* = 8.2, 5.7 Hz, CH_ar_, 2H), 4.49 (s, OH, 2H), 2.64 (q, *J* = 7.6 Hz, CH_2_, 2H), 2.58 (t, *J* = 7.8 Hz, CH_2_, 2H), 1.65 – 1.58 (m, CH_2_, 2H), 1.37 – 1.31 (m, CH_2_-CH_2_,4H), 1.24 (t, *J* = 7.6 Hz, CH_3_, 3H), 0.90 (t, *J* = 7.0 Hz, CH_3_, 3H). ^13^C NMR (126 MHz, CDCl_3_) δ 150.80 (C_ar_-O), 150.76 (C_ar_-O), 137.30 (C_ar_), 136.04 (C_ar_), 130.93 (C_ar_), 130.40 (C_ar_), 129.68 (C_ar_), 129.15 (C_ar_), 123.74 (C_ar_), 123.65 (C_ar_), 116.51 (C_ar_), 116.42 (C_ar_), 35.04 (ar-CH_2_), 31.50 (ar-CH_2_), 31.35 (CH_2_), 27.98 (CH_2_), 22.52 (CH_2_), 15.77 (CH_3_), 14.02 (CH_3_). LC/ESI-MS (positive mode) m/z 285 (M+H)^+^_,_ (negative mode) m/z 283 (M-H)^-^, 100%. Yield 27.2%.

**5-Ethyl-5'-hexylbiphenyl-2,2'-diol (57)** ^1^H NMR (500 MHz, CDCl_3_) δ 7.15 – 7.07 (m, CH_ar_, 4H), 6.94 (dd, *J* = 8.2, 6.0 Hz, CH_ar_, 2H), 5.33 (s, OH, 2H), 2.64 (q, *J* = 7.6 Hz, CH_2_, 2H), 2.58 (t, *J* = 7.8 Hz, CH_2_, 2H), 1.61 (m, CH_2_, 2H), 1.37 – 1.28 (m, CH_2_-CH_2_-CH_2_, 6H), 1.24 (t, *J* = 7.6 Hz, CH_3_, 3H), 0.89 (t, *J* = 7.0 Hz, CH_3_, 3H). ^13^C NMR (126 MHz, CDCl_3_) δ 150.76 (C_ar_-O), 150.72 (C_ar_-O), 137.33 (C_ar_), 136.07 (C_ar_), 130.92 (C_ar_), 130.40 (C_ar_), 129.69 (C_ar_), 129.16 (C_ar_), 123.68 (C_ar_), 123.59 (C_ar_), 116.49 (C_ar_), 116.41 (C_ar_), 35.07 (ar-CH_2_), 31.69 (ar-CH_2_), 31.63 (CH_2_), 28.97 (CH_2_), 27.98 (CH_2_), 22.60 (CH_2_), 15.77 (CH_3_), 14.07 (CH_3_). LC/ESI-MS (positive mode) m/z 299 (M+H)^+^_,_ (negative mode) m/z 297 (M-H)^-^, 99.3%. Yield 28.0%.

**5-Butyl-5'-propylbiphenyl-2,2'-diol (58)** ^1^H NMR (500 MHz, CDCl_3_) δ 7.12 (dd, *J* = 8.2, 1.9 Hz, CH_ar_, 2H), 7.08 (d, *J* = 2.1 Hz, CH_ar_, 2H), 6.94 (dd, *J* = 8.2, 1.4 Hz, CH_ar_, 2H), 5.54 (s, OH, 2H), 2.61 – 2.55 (m, ar-CH_2_, 4H), 1.69 – 1.56 (m, CH_2_-CH_2_, 4H), 1.37 (m, CH_2_, 2H,), 0.96 (t, *J* = 5.5 Hz, CH_3_, 3H), 0.93 (t, *J* = 5.5 Hz, CH_3_, 3H). ^13^C NMR (126 MHz, CDCl_3_) δ 150.74 (C_ar_-O), 150.70 (C_ar_-O), 136.00 (C_ar_), 135.79 (C_ar_), 130.95 (C_ar_), 130.89 (C_ar_), 129.73 (C_ar_), 129.68 (C_ar_), 123.52 (C_ar_, 2C), 116.38 (C_ar_, 2C), 37.13 (ar-CH_2_), 34.73 (ar-CH_2_), 33.80 (CH_2_), 24.69 (CH_2_), 22.32 (CH_2_), 13.91 (CH_3_), 13.79 (CH_3_). Mp 95-96 °C. LC/ESI-MS (negative mode) m/z 283 (M-H)^-^_,_ 99.9%. Yield 27.9%.

**5-*Tert*-butyl-5'-propylbiphenyl-2,2'-diol (59)** ^1^H NMR (500 MHz, CDCl3) δ 7.34 (dd, *J* = 8.5, 2.5 Hz, Ch_ar_, 1H), 7.26 (d, *J* = 2.5 Hz, Ch_ar_, 1H), 7.14 (dd, *J* = 8.2, 2.2 Hz, Ch_ar_, 1H), 7.08 (d, *J* = 2.2 Hz, Ch_ar_, 1H), 6.96 (dd, *J* = 8.4, 4.8 Hz, Ch_ar_, 2H), 5.37 (s, OH, 2H), 2.59 – 2.56 (t, *J* = 7.8 Hz, ar-CH_2_, 2H), 1.68 – 1.60 (m, CH_2_, 2H), 1.33 (s, C(CH_3_)_3_, 9H), 0.96 (t, *J* = 7.3 Hz, CH_3_, 3H). ^13^C NMR (126 MHz, CDCl3) δ 150.88 (C_ar_-O), 150.58 (C_ar_-O), 144.37 (C_ar_), 135.82 (C_ar_), 130.96 (C_ar_), 129.82 (C_ar_), 127.93 (C_ar_), 126.90 (C_ar_), 123.61 (C_ar_), 122.89 (C_ar_), 116.38 (C_ar_), 116.09 (C_ar_), 37.16 (ar-CH_2_), 34.23 (ar-C), 31.53 ((CH_3_)_3_), 24.72 (CH_3_), 13.83 (CH_3_). Mp 161-162 °C. LC/ESI-MS (positive mode) m/z 283 (M+H)^+^_,_ (negative mode) m/z 285 (M-H)^-^_,_ 98.3%. Yield 27.0%.

**5-Heptyl-5'-propylbiphenyl-2,2'-diol (62)** ^1^H NMR (500 MHz, CDCl_3_) δ 7.12 (dd, *J* = 8.2, 2.2 Hz, CH_ar_, 2H), 7.07 (d, *J* = 2.2 Hz, CH_ar_, 2H), 6.94 (dd, *J* = 8.2, 1.9 Hz, CH_ar_, 2H), 5.40 (s, OH, 2H), 2.62 – 2.54 (m, ar-CH_2_, 4H), 1.69 – 1.57 (m, CH_2_, 4H), 1.39 – 1.24 (m, CH_2_-CH_2_-CH_2_-CH_2_, 8H), 0.95 (t, *J* = 7.3 Hz, CH_3_, 3H), 0.88 (t, *J* = 7.0 Hz, CH_3_, 3H). ^13^C NMR (126 MHz, CDCl_3_) δ 150.79 (C_ar_-O), 150.74 (C_ar_-O), 136.06 (C_ar_), 135.79 (C_ar_), 130.92 (C_ar_), 130.85 (C_ar_), 129.78 (C_ar_), 129.73 (C_ar_), 123.38 (C_ar_, 2C), 116.37 (C_ar_, 2C), 37.14 (ar-CH_2_), 35.04 (ar-CH_2_), 31.79 (CH_2_), 31.65 (CH_2_), 29.24 (CH_2_), 29.13 (CH_2_), 24.69 (CH_2_), 22.63 (CH_2_), 14.06 (CH_3_), 13.79 (CH_3_). LC/ESI-MS (negative mode) m/z 325 (M-H)^-^_,_ 100%. Yield 28.1%.

**5-Octyl-5'-propylbiphenyl-2,2'-diol (63)** ^1^H NMR (500 MHz, CDCl_3_) δ 7.12 (dd, *J* = 8.3, 2.2 Hz, Ch_ar_, 2H), 7.07 (d, *J* = 1.9 Hz, Ch_ar_, 2H), 6.94 (dd, *J* = 8.2, 1.7 Hz, Ch_ar_, 2H), 5.42 (s, OH, 2H), 2.57 (m, ar-CH_2_, 4H), 1.70 – 1.56 (m, CH_2_, 4H), 1.37 – 1.22 (m, CH_2_-CH_2_-CH_2_-CH_2_-CH_2_, 10H), 0.96 (t, *J* = 7.3 Hz, CH_3_, 3H), 0.88 (t, *J* = 6.9 Hz, CH_3_, 3H). ^13^C NMR (126 MHz, CDCl_3_) δ 150.79 (C_ar_-O), 150.74 (C_ar_-O), 136.08 (C_ar_), 135.80 (C_ar_), 130.94 (C_ar_), 130.87 (C_ar_), 129.79 (C_ar_), 129.73 (C_ar_), 123.42 (C_ar_), 116.39 (C_ar_), 37.15 (ar-CH_2_), 35.06 (ar-CH_2_), 31.86 (CH_2_), 31.66 (CH_2_), 29.44 (CH_2_), 29.30 (CH_2_), 29.25 (CH_2_), 24.70 (CH_2_), 22.64 (CH_2_), 14.07 (CH_3_), 13.80 (CH_3_). LC/ESI-MS (positive mode) m/z 341 (M+H)^+^_,_ (negative mode) m/z 339 (M-H)^-^_,_ 100%. Yield 26.4%.

**5-Butyl-5'-pentylbiphenyl-2,2'-diol (64)** ^1^H NMR (500 MHz, CDCl_3_) δ 7.12 (dd, *J* = 8.2, 2.2 Hz, Ch_ar_, 2H), 7.08 (d, *J* = 2.1 Hz, Ch_ar_, 2H), 6.94 (d, *J* = 8.2 Hz, Ch_ar_, 2H), 4.91 (s, OH, 2H), 2.62 – 2.56 (m, ar-CH_2_, 4H), 1.65 – 1.56 (m, CH_2_ 4H), 1.41 – 1.28 (m, CH_2_-CH_2_-CH_2_, 6H), 0.93 (t, *J* = 7.4 Hz, CH_3_, 3H), 0.90 (t, *J* = 7.0 Hz, 3H). ^13^C NMR (126 MHz, CDCl3) δ 150.78 ((C_ar_-O), 2C), 136.04 ((C_ar_), 2C), 130.89 ((C_ar_), 2C), 129.71 ((C_ar_), 2C), 123.52 ((C_ar_), 2C), 116.42 ((C_ar_), 2C), 35.03 (ar-CH_2_), 34.75 (ar-CH_2_), 33.82 (CH_2_), 31.50 (CH_2_), 31.35 (CH_2_), 22.52 (CH_2_), 22.34 (CH_2_), 14.03 (CH_3_), 13.94 (CH_3_). LC/ESI-MS (negative mode) m/z 311 (M-H)^-^_,_ 100%. Yield 23.9%.

**5-Butyl-5'-hexylbiphenyl-2,2'-diol (65)** ^1^H NMR (500 MHz, CDCl_3_) δ 7.12 (dd, *J* = 8.3, 1.8 Hz, Ch_ar_, 2H), 7.07 (d, *J* = 2.1 Hz, Ch_ar_, 2H), 6.94 (d, *J* = 8.2 Hz, Ch_ar_, 2H), 5.41 (s, OH, 2H), 2.66 – 2.48 (m, ar-CH_2_, 4H), 1.65 – 1.56 (m, CH_2_, 4H), 1.42 – 1.26 (m, CH_2_-CH_2_-CH_2_-CH_2_, 8H), 0.93 (t, *J* = 7.4 Hz, CH_3_, 3H), 0.89 (t, *J* = 7.0 Hz, CH_3_, 3H). ^13^C NMR (126 MHz, CDCl3) δ 150.72 ((C_ar_-O), 2C), 136.04 ((C_ar_), 2C), 130.84 ((C_ar_), 2C), 129.71 ((C_ar_), 2C), 123.38 ((C_ar_), 2C), 116.36 ((C_ar_), 2C), 35.03 (ar-CH_2_), 34.71 (ar-CH_2_), 33.78 (CH_2_), 31.66 (CH_2_), 31.59 (CH_2_), 28.93 (CH_2_), 22.56 (CH_2_), 22.31 (CH_2_), 14.03 (CH_3_), 13.89 (CH_3_). LC/ESI-MS (positive mode) m/z 327 (M+H)^+^_,_ (negative mode) m/z 325 (M-H)^-^_,_ 100%. Yield 27.2%.
