# Supplementary material for: The Natural Product Magnolol as a Lead Structure for the Development of Potent Cannabinoid Receptor Agonists
Source: PLoS One. 2013 Oct 30;8(10):e77739. doi: 10.1371/journal.pone.0077739 (PMC3813752; doi:10.1371/journal.pone.0077739)
Supplement: Dataset S2 — Alignment of amino acid sequences. Applied Software: Clustal W2 provided by European Molecular Biology Laboratory - European Bioinformatics Institute (EMBL-EBI) (http://www.ebi.ac.uk/Tools/msa/clustalw2/). (DOCX) [file pone.0077739.s014.docx]

GPR18 Gene ID: 2841

GPR55 Gene ID: 9290

CB_1_ Gene ID: 1268 isoform a

CB_2_ Gene ID: 1269

GPR18 and GPR55 were aligned for their identity with the amino acid sequence of cannabinoid receptors 1 and 2.

GPR18 amino acid identity with CB_1_ 12%

GPR18 amino acid identity with CB_2_ 7%

GPR55 amino acid identity with CB_1_ 15%

GPR55 amino acid identity with CB_2_ 13%
